# Exact and efficient phylodynamic simulation from arbitrarily large populations

**Published:** 2024-02-27

**Authors:** Michael Celentano, William S. DeWitt, Sebastian Prillo, Yun S. Song

**Affiliations:** 1 Department of Statistics, University of California, Berkeley; 2 Computer Science Division, University of California, Berkeley

## Abstract

Many biological studies involve inferring the genealogical history of a sample of individuals from a large population and interpreting the reconstructed tree. Such an ascertained tree typically represents only a small part of a comprehensive population tree and is distorted by survivorship and sampling biases. Inferring evolutionary parameters from ascertained trees requires modeling both the underlying population dynamics and the ascertainment process. A crucial component of this phylodynamic modeling involves tree simulation, which is used to benchmark probabilistic inference methods. To simulate an ascertained tree, one must first simulate the full population tree and then prune unobserved lineages. Consequently, the computational cost is determined not by the size of the final simulated tree, but by the size of the population tree in which it is embedded. In most biological scenarios, simulations of the entire population are prohibitively expensive due to computational demands placed on lineages without sampled descendants. Here, we address this challenge by proving that, for any partially ascertained process from a general multi-type birth-death-mutation-sampling (BDMS) model, there exists an equivalent *pure birth* process (i.e., no death) with mutation and *complete sampling*. The final trees generated under these processes have exactly the same distribution. Leveraging this property, we propose a highly efficient algorithm for simulating trees under a general BDMS model. Our algorithm scales linearly with the size of the final simulated tree and is independent of the population size, enabling simulations from extremely large populations beyond the reach of current methods but essential for various biological applications. We anticipate that this unprecedented speedup will significantly advance the development of novel inference methods that require extensive training data.

## 1 Introduction

Phylogenetic trees describe the ancestral relationships within a sample of individuals from a large population and are central objects in studies of evolution.^1^ These individuals can represent extant or extinct species in macroevolution [16,18], viral sequences collected over time in epidemiology [29], or individual cells in studies of cancer evolution or affinity maturation [5, 7, 8, 26, 35], for example. The branch lengths and branching rates in these phylogenies have been used to infer shifts in diversification rates across lineages and over time in the tree of life [4, 6, 14], estimate effective reproduction number or predict future strains in viral evolution [23, 30], and estimate rates of metastasis, phylodynamics, plasticity, or paths of tumor evolution in cancer [34], among many other applications.

Due to death and incomplete sampling in these applications, the inferred phylogeny typically represents a partial history of the full population. This partial observation process can lead to bias in the estimation of fundamental population parameters. For example, the branching rate in the observed phylogeny is typically substantially smaller than that in the full population phylogeny [17, 20]. Moreover, certain population-level quantities may not be identified by the distribution of the observed phylogeny without further assumptions [12–14].

Simulations play a central role in tree-based inference. Their uses include benchmarking existing methods [26, 32], training novel methods based on simulated data [31], and, in some approaches, may form a sub-routine of the inferential method itself [33]. In order to faithfully represent the relationship between population-level parameters and the observed data, these simulations must include the partial observation process. Current approaches simulate the partial observation process directly. First, the phylogeny of the full population is simulated; next, some lineages are sampled; and finally, lineages that are not ancestral to a sampled lineage are pruned from the full phylogeny [20].

In many applications, full-population simulations are prohibitively expensive because they must expend substantial computational resources on lineages that, because they have no sampled descendants, do not appear in the observed phylogeny. For example, in a typical year, there are tens of millions of flu infections in the United States alone [27]. Nevertheless, large phylogenetic analyses of flu are often limited to tens of thousands of sampled viral sequences [1], and it is not uncommon to analyze phylogenies with only a few hundred samples per year [21]. In cancer, a cubic centimeter of tumor mass contains roughly one billion cells, yet single-cell resolution studies of cancer with CRISPR-Cas9-based lineage tracing assays sequence fewer than 100,000 cells [10, 25, 28, 34], representing roughly 0.01% of the population. Recent works at best simulate a population of 40, 000 cancer cells subsequently sub-sampled down to 1% [10, 24], which is highly unrealistic.

This paper proposes a novel algorithm for exact (up to numerical and time-discretization error) simulation from a general class of multi-type birth-death processes with birth, death, mutation, and incomplete sampling. This class of models agrees with those considered by MacPherson et al. [15], which unified a collection of models used in a wide range of biological contexts, including the applications described above [4, 6, 11, 14, 16, 18, 19, 22, 30]. This general class of models is referred to as “birth-death-mutation-sampling” (BDMS) models. The computational cost of our algorithm for simulating BDMS models scales not with the size of the full population, as do existing approaches, but rather with the sample size. It thus avoids expending computational resources on unobserved lineages and is essentially optimal in terms of runtime. Based on the typical numbers for flu and cancer evolution studies described above, our approach can reduce computation by a factor of 1,000 to 10,000, and make it feasible to simulate with realistic population-size and sub-sampling parameters. Thus, our novel algorithm will facilitate the assessment of existing methods under more biologically realistic settings and enable the development of simulation-based training and estimation approaches.

From a technical point of view, our algorithm is based on the following insight. The distribution of the observed phylogeny in any BDMS model is equivalent to that generated by an alternative BDMS model with no death and complete sampling. In this alternative BDMS model, which we call the *forward-equivalent model*, the full phylogeny corresponds to the observed phylogeny. Therefore, simulating from the forward-equivalent model allows us to avoid expending computational resources on unobserved lineages while generating observed trees from the same distribution.

Our insight is closely related to recent work on statistical identifiability in sub-sampled phylogenetic birth-death models [12–14]. This work observed that in the single-type setting, multiple population-level parameterizations give rise to the same observed data distribution. Our algorithm is based on the lack of identifiability in the more general multi-type setting. Whereas lack of identifiability prohibits exact inference (without further assumptions), it also facilitates simulation. Because multiple BMDS models give rise to the same observed data distribution, we can base our simulations on those that can be simulated most efficiently.

## 2 The birth-death-mutation-sampling model

The BDMS model we study is a multi-type birth-death process with mutations and incomplete sampling as presented by MacPherson et al. [15]. It consists of a collection of lineages that give birth, die, mutate, and are sampled over time. The model begins with a single lineage at time $\tau =t_{\max}>0$ measured as the (positive) distance in time before the present day, τ=0. The single lineage at time $\tau =t_{\max}>0$ is initialized with type a drawn from a distribution $\pi =\left(\pi_{1},\ldots ,\pi_{d}\right)$ over a finite collection of d types indexed 1,…,d. Each lineage progresses independently of all other lineages, with events arriving according to Poisson point processes with potentially time-varying intensity. These processes are as follows. At time τ, a lineage of type a gives birth to a lineage of type b at rate $\lambda_{a,b}\left(\tau \right)$, dies at rate $\mu_{a}\left(\tau \right)$, and is sampled at rate $\psi_{a}\left(\tau \right)$. Upon sampling, a lineage dies with probability $r_{a}\left(\tau \right)\in \left[0,1\right]$. When a lineage gives birth, there are two daughter lineages, one with type a and one with type b. The parameter $\lambda_{a,a}\left(\tau \right)$ describes the rate at which births without mutation occur. For b≠a, the parameter $\lambda_{a,b}\left(\tau \right)$ describes the rate of cladogenetic mutation. Anagenetic mutations—ones which occur at non-birth events—from type a to type b occur at rate $\gamma_{a,b}\left(\tau \right)$, which is non-zero for only b≠a.

In addition to the above processes, whose events arrive according to time-dependent Poisson point processes, we allow for concerted sampling events (CSEs) [15]. These are instances in time at which a fraction of the population gets simultaneously sampled. These occur at fixed times $t_{0}=0<t_{1}<\cdots <t_{L}\leq t_{\max}$. The $l^{\text{th}}$ CSE consists of sampling all extant lineages of type a at time $t_{l}$ independently with probability $\rho_{a,l}$. During a CSE, each sampled lineage dies with probability $r_{a,l}$. The model is defined by the set of parameters $\left(\pi ,\lambda_{a,b},\mu_{a},\psi_{a},r_{a},\gamma_{a,b},\rho_{a,b},r_{a,l}\right)$, whose interpretations are summarized in Table 1.

**Figure 1:**
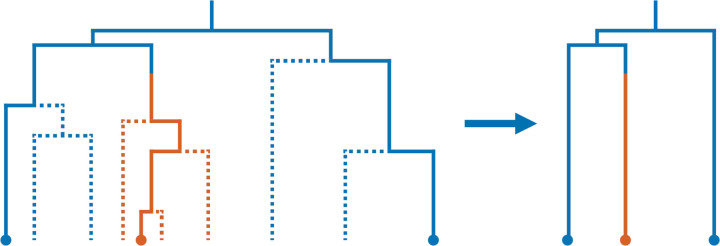
Diagram of partial observation process. The full phylogeny is displayed on the left. Color represents type, with one anagenetic mutation event shown. All sampling events occur at the present day, represented by solid circles at the leaves. Lineages that are not ancestral to a sampled lineage are shown in dashed lines. The observed phylogeny is shown on the right.

The reconstructed phylogeny refers to the subset of the full phylogeny containing only those lineages ancestral to a sampled lineage [20]. In Figure 1, we provide a diagram demonstrating the partial observation process on a full phylogeny and the corresponding reconstructed phylogeny. In applications, it is typically the reconstructed phylogeny (or an estimate of it based on sequence data) that is observed. Thus, the distribution of the reconstructed phylogeny and its corresponding likelihood in terms of model parameters, rather than the distribution and likelihood for the full phylogeny, must be used as the basis for inference. A large body of work derives the likelihood for reconstructed phylogenies as a solution to a system of ordinary differential equations which can be solved numerically [11,16,19]. Our simulation method will also rely on solving a system of ordinary differential equations.

## 3 Simulation algorithms

We describe two methods for simulating reconstructed phylogenies. The first, which we call the *full simulation*, simulates the full phylogeny and then removes (or “prunes”) lineages that are not ancestral to a sampling event; this is the current state-of-the-art. The second is our novel method, which we call the *forward-equivalent simulation*; this approach simulates only those lineages that will have sampled descendants, thereby greatly reducing computational resources for most practical applications.

### 3.1 The full simulation

There are a variety of methods for simulating the full phylogeny which differ according to whether they grow the phylogeny in a depth-first or breadth-first manner and in how they determine the time and type of birth, death, mutation, and sampling events. We used an efficient breadth-first implementation [3] whose time complexity is linear in the number of simulated events, where a single event corresponds to a birth, death, mutation, or sampling event in the simulated population. Pseudocode for the algorithm we used is stated in Algorithm 1. The contribution of this paper is the forward equivalent simulation described in the next section, which can be viewed as a wrapper around the full simulation presented here. Indeed, the forward equivalent simulation can be wrapped around any implementation of a time-varying BDMS model. The implementation we used was selected to optimize time complexity in the number of simulated events. While it is not the contribution of this paper, it is presented for completeness.


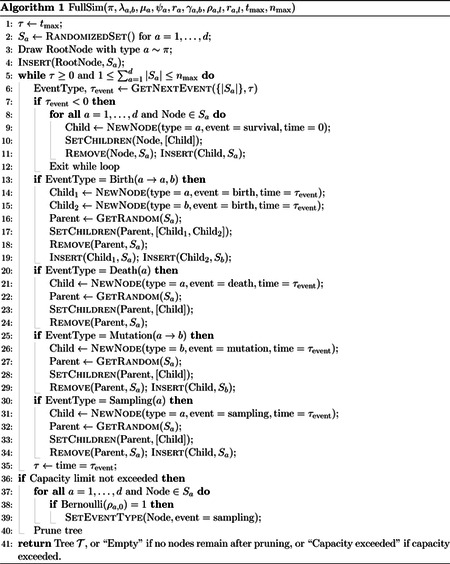


In words, Algorithm 1 simulates forward in time starting at $\tau =t_{\max}$ and ending at τ=0 or until all lineages in the simulation die or a user-specified capacity limit $n_{\max}$ is exceeded. The nodes in the tree correspond to birth, death, mutation, or sampling events. As we grow the tree, each node contains information about its type, event type, and time.

We generate the tree in a breadth-first manner. At each moment, the algorithm maintains biological time τ and the tree generated up until time τ. Each iteration generates the next event in biological time anywhere in the tree, and advances the tree to the time of that next event. Thus, consecutive iterations generate events that are consecutive in biological time but which need not occur to the same lineage or be close phylogenetically.

In order to achieve this, at each iteration the algorithm maintains, for each type a=1,…,d, a set $S_{a}$ containing the most recent event nodes for all extant lineages of type a. The next event is then generated as follows:

1. First the time and type of the next event are determined. The type of the next event specifies whether it is a birth, death, mutation, or sampling event, the type of the parent node, and, for mutation events, the type of the child or children nodes. It does not specify on which lineage the event occurs. In Algorithm 1, this step is represented by the function GetNextEvent. The time and type of the next event can be determined using the model parameters $\left(\pi ,\lambda_{a,b},\mu_{a},\psi_{a},r_{a},\gamma_{a,b},\rho_{a,b},r_{a,l}\right)$, the sizes of the extant populations for each type ${\left\{\left|S_{a}\right|\right\}}_{a=1}^{d}$, and the current biological time τ. In Appendix A, we describe an implementation of GetNextEvent which runs in constant time in the size of the extant population.
2. The parent node of the next event node is determined. If the event type returned by GetNextEvent specifies that the parent is a type a, we pick a parent node uniformly at random from $S_{a}$. As we describe below, we use a data structure for $S_{a}$ that permits uniform random sampling in $O\left(1\right)$ time.
3. The phylogeny is advanced to the time of the next event. This involves creating a new event node whose parent is as chosen in the previous step and updating the sets $\left\{S_{a}\right\}$ as needed. See Algorithm 1 for details (which depend on the event type). This step requires possibly inserting or removing nodes from the sets $S_{a}$. As we describe below, we use a data structure for $S_{a}$ that permits insertion and removal in constant time.

These iterations progress until either the time of the next event is after present time τ=0 or a user-specified population size capacity limit is exceeded. If the time of the next event is after the present time, then this event does not occur. Instead, all extant lineages are advanced to the present, and sampling occurs for each lineage according to the sampling probabilities $\rho_{a,0}$.

Note that we allow for a user-specified capacity limit even though this is not formally a part of the BDMS model. Practically, it is useful to have an implementation that allows termination based on a user-specified capacity limit. Depending on the model parameters, the population size can grow exponentially with time. Thus, if $t_{\max}$ is set too large, the algorithm will fail to terminate in a reasonable amount of time. In practice, one chooses model parameters such that simulation to the present is, except in rare cases, feasible with available computational resources. The capacity limit is chosen large enough so that it rarely is reached but serves as protection against exponential blow-up. In this case, the capacity limit will have a negligible impact on the distribution of simulated trees.

After the full phylogeny has been simulated as described above, the tree is pruned to remove all lineages and nodes that are not ancestral to a sampling event. Moreover, birth events in which only one child lineage survives are removed. The pruning operation can be carried out in time linear in the number of events in the full phylogeny.

For simplicity and conciseness, we have omitted from this description and from the pseudo-code in Algorithm 1 concerted sampling events (CSEs) except for the one at the present day. These are straightforward to add and do not affect our discussion on computational complexity. Indeed, when the time of the next event is determined to be after the next concerted sampling event, the event does not occur. Instead, all extant lineages are advanced to the time of the concerted sampling event, at which point the appropriate sampling and death events are carried out.

We implement Algorithm 1 so that each iteration of the while loop (which corresponds to a single event) runs in constant time in the population size. This requires that GetNextEvent has constant time complexity $O\left(1\right)$ in the population size, and the sets $S_{a}$ are implemented using a data structure which supports insertion, removal, and uniform random sampling in constant time in the population size. We describe these implementation details in Appendix A. With this implementation, Algorithm 1 has time complexity linear in the number of events in the full phylogeny. In Section 4 and Supplementary Figure 2, we empirically validate that the computation time scales linearly with the number of events in the full phylogeny.

As we describe in Appendix A, the time complexity of GETNEXTEVENT is quadratic in the size of the type space d. Thus, the overall runtime of Algorithm 1 when taking into account the size of the type space is $O\left(\#\text{nodes}\left(T\right)d^{2}\right)$ where $\#\text{nodes}\left(T\right)$ is the number of events in the full phylogeny T. The quadratic factor $d^{2}$ may be replaced by $d+\log\left(\#\text{nodes}\left(T\right)\right)$ by using an alternative implementation based on a priority queue. For applications with large type spaces, this implementation may be preferred. We describe this approach in Appendix A.4. In this work, however, we focus on the approach provided in Algorithm 1, since its runtime is strictly linear in the number of events in the full phylogeny.

### 3.2 The forward-equivalent simulation

The full simulation expends computational time on all lineages, including those that have no sampled descendants and thus do not appear in the reconstructed phylogeny. We propose a method that does not expend computational resources on such unobserved lineages. Avoiding such computation is non-trivial: the appearance of a lineage in the reconstructed phylogeny depends on the occurrence (or not) of a future sampling event. Thus, in Algorithm 1, the appearance of an event in the reconstructed phylogeny cannot be determined at the time the event is simulated.

In this section, we show that the distribution of the reconstructed phylogeny in the full model conditional on it being non-empty is equal to that generated by an alternative BDMS model without death and with complete sampling at the present. We call this alternative BDMS model the *forward-equivalent model*.

Precisely, consider any set of model parameters $\text{Θ}:=\left(\pi ,\lambda_{a,b},\mu_{a},\psi_{a},r_{a},\gamma_{a,b},\rho_{a,l},r_{a,l}\right)$ for the full simulation. Our main result is that, for any Θ, there is an alternative set of parameters ${\text{Θ}}^{\text{FE}}:=\left(\pi^{\text{FE}},\lambda_{a,b}^{\text{FE}},\mu_{a}^{\text{FE}},\psi_{a}^{\text{FE}},r_{a}^{\text{FE}},\gamma_{a,b}^{\text{FE}},\rho_{a,l}^{\text{FE}},r_{a,l}^{\text{FE}}\right)$ satisfying $\mu_{a}^{\text{FE}}\left(\tau \right)=0$ for all $a\in \left\{1,\ldots ,d\right\}$, $\tau \in \left[0,t_{\max}\right]$ (no death) and $\rho_{a,0}^{\text{FE}}=1$ (complete sampling at the present) such that the distribution of the reconstructed phylogeny T under Θ conditional on being non-empty, T≠T0, is equal to the distribution of the reconstructed phylogeny under ${\text{Θ}}^{\text{FE}}$ (with T0 denoting the empty tree realization, which corresponds to a full tree in which all lineages are either extinct or unsampled, so that pruning leaves no ascertained sub-tree). The parameters ${\text{Θ}}^{\text{FE}}$ are given precisely in Section 3.3. Denote the reconstructed phylogeny in the full model by T and the reconstructed phylogeny (which is equivalent to the full phylogeny) in the forward-equivalent model by $T^{\text{FE}}$.

**Theorem 1.** The distribution of the reconstructed phylogeny in the full model conditional on its non-emptiness is equivalent to the unconditional distribution of the reconstructed/full phylogeny in the forward-equivalent model:

(1) LawT|T≠T0=LawTFE.

We prove Theorem 1 in Appendix B.

Theorem 1 suggests simulating the reconstructed phylogeny in the full model by running the full simulation on the parameters $\left(\pi^{\text{FE}},\lambda_{a,b}^{\text{FE}},\mu_{a}^{\text{FE}},\psi_{a}^{\text{FE}},r_{a}^{\text{FE}},\gamma_{a,b}^{\text{FE}},\rho_{a,l}^{\text{FE}},r_{a,l}^{\text{FE}}\right)$. This approach, which we call the forward-equivalent simulation, is given in Algorithm 2. It generates reconstructed phylogenies from the distribution LawT|T≠T0. If one wants to generate reconstructed phylogenies from $\text{Law}\left(T\right)$ allowing for emptiness, one can first generate a Bernoulli random variable B with success probability ℙT≠T0 (which is computed in Section 3.3) and, if B=0, return T0, whereas if B=1, return $\text{FESim}\left(\pi ,\lambda_{a,b},\mu_{a},\psi_{a},r_{a},\gamma_{a,b},\rho_{a,l},r_{a,l}\right)$. In practice, one often will want to generate non-empty phylogenies, so this extra step will not be necessary.


$$\bar{\underline{\begin{array}{l} \text{Algorithm }2\text{ FESim}\left(\pi ,\lambda_{a,b},\mu_{a},\psi_{a},r_{a},\gamma_{a,b},\rho_{a,l},r_{a,l},t_{\max}\right)\\ \bar{\;\text{return FullSim}\left(\pi^{\text{FE}},\lambda_{a,b}^{\text{FE}},\mu_{a}^{\text{FE}}=0,\psi_{a}^{\text{FE}},r_{a}^{\text{FE}},\gamma_{a,b}^{\text{FE}}{\left(\rho_{a,l}^{\text{FE}}\right)}_{l\geq 1},\rho_{a,0}^{\text{FE}}=1,r_{a,l}^{\text{FE}},t_{\max}\right)} \end{array}}}$$

In the forward-equivalent model, all lineages are observed and the full and the reconstructed phylogeny are the same. When used to simulate reconstructed phylogenies in models with death or incomplete sampling, the forward-equivalent simulation effectively avoids expending computational resources on lineages that are not observed. This leads to substantial computational savings when either only a small fraction of the full phylogeny is observed or the full phylogeny only rarely survives until the present. An analysis of computational complexity is carried out in Section 3.4.

Implementing Algorithm 2 requires access to the forward-equivalent model parameters ${\text{Θ}}^{\text{FE}}$. These can be precomputed once, and then Algorithm 2 can be used to generate an arbitrary number of trees. These precomputations are described in Appendix A. The cost of precomputation is amortized over the number of simulated trees, so contributes negligible computational cost when this number is large. In our experiments, pre-computations were very fast.

### 3.3 The forward-equivalent model parameters

In this section, we define the parameters ${\text{Θ}}^{\text{FE}}$ explicitly in terms of the parameters Θ of the full model. A key quantity is the non-observation probability $E_{a}\left(\tau \right)$ for a=1,…,d and $\tau \in \left[0,t_{\max}\right]$ in the full model. It is the probability that, given a lineage of type a in the population at time τ, none of its descendants will be sampled. At CSEs, the non-observation probability will be different immediately prior to and after the realization of the CSE’s sampling and death events. We denote the non-observation probability prior to the realization of these events by $E_{a}\left(t_{l}^{+}\right)$ and after the realization of these events by $E_{a}\left(t_{l}^{-}\right)$, where $t_{l}$ is the time of the CSE. As shown in [15, Eq. (15)]), the non-observation probabilities are given by

(2) $$\begin{array}{l} \text{Initialization: }E_{a}\left(0^{-}\right)=1,\\ \text{Between}\;\text{CSEs: }\frac{dE_{a}\left(\tau \right)}{\text{d}\tau }=\sum_{b=1}^{d}\lambda_{a,b}\left(\lambda \right)\left[E_{a}\left(\tau \right)E_{b}\left(\tau \right)-E_{a}\left(\tau \right)\right]\\ \;+\mu_{a}\left(\tau \right)\left[1-E_{a}\left(\tau \right)\right]\\ \;+\psi_{a}\left(\tau \right)\left[0-E_{a}\left(\tau \right)\right]\\ \;+\sum_{b=1}^{d}\gamma_{a,b}\left(\tau \right)\left[E_{b}\left(\tau \right)-E_{a}\left(\tau \right)\right],\\ \text{At}\;\text{CSEs: }E_{a}\left(t_{l}^{-}\right)=\underset{\tau ↑t_{l}}{\lim}E_{a}\left(\tau \right),\;E_{a}\left(t_{l}^{+}\right)=\left(1-\rho_{a,l}\right)E_{a}\left(t_{l}^{-}\right). \end{array}$$

For completeness, we derive (2) in Appendix B. The non-observation probability has previously played an important role in likelihood-based and Bayesian methods of inference based on the reconstructed phylogeny [4, 6, 15, 16, 18, 23]. The forward-equivalent model parameters are given by

(3) $$\begin{array}{l} \;\pi_{a}^{\text{FE}}=\frac{\pi_{a}\left[1-E_{a}\left(t_{\max}\right)\right]}{\sum_{b}\pi_{b}\left[1-E_{b}\left(t_{\max}\right)\right]},\;\lambda_{a,b}^{\text{FE}}\left(\tau \right)=\left[1-E_{b}\left(\tau \right)\right]\lambda_{a,b}\left(\tau \right),\\ \mu_{a}^{\text{FE}}\left(\tau \right)=0,\;\psi_{a}^{\text{FE}}\left(\tau \right)=\frac{\psi_{a}\left(\tau \right)}{1-E_{a}\left(\tau \right)},\\ r_{a}^{\text{FE}}\left(\tau \right)=r_{a}\left(\tau \right)+\left[1-r_{a}\left(\tau \right)\right]E_{a}\left(\tau \right),\;\gamma_{a,b}^{\text{FE}}\left(\tau \right)=\frac{1-E_{b}\left(\tau \right)}{1-E_{a}\left(\tau \right)}\left[\gamma_{a,b}\left(\tau \right)+E_{a}\left(\tau \right)\lambda_{a,b}\left(\tau \right)\delta_{a\neq b}\right],\\ \;\rho_{a,l}^{\text{FE}}=\frac{\rho_{a,l}}{1-E_{a}\left(t_{l}^{+}\right)},\;r_{a,l}^{\text{FE}}=r_{a,l}+\left(1-r_{a,l}\right)E_{a}\left(t_{l}^{-}\right). \end{array}$$

By (2), $E_{a}\left(0^{+}\right)=1-\rho_{a,0}$, whence by (3), $\rho_{a,0}^{\text{FE}}=1$. Thus, in the forward-equivalent simulation, all lineages are sampled at the present τ=0.

It is enlightening to interpret some of the equations in (3). The equation for $\lambda_{a,b}^{\text{FE}}\left(\tau \right)$ indicates that the birth rate in the reconstructed phylogeny is smaller than that in the population. This effect is stronger for individuals that are more unlikely to have sampled descendants. The equation for $\gamma_{a,b}^{\text{FE}}\left(\tau \right)$ indicates that anagenetic mutation rates can be upward or downward biased in the reconstructed phylogeny. In particular, mutations from types that are unlikely to have sampled descendants and to types that are likely to have sampled descendants are higher in the reconstructed phylogeny than in the population; mathematically, $E_{a}\left(\tau \right)>E_{b}\left(\tau \right)$ implies $\frac{1-E_{b}\left(\tau \right)}{1-E_{a}\left(\tau \right)}>1$, so $\gamma_{a,b}^{\text{FE}}\left(\tau \right)>\gamma_{a,b}\left(\tau \right)$. One factor that increases the probability of having a sampled descendant is having “higher fitness” in terms of a larger birth rate or lower death rate, leading to a higher chance of survival. Thus, these equations indicate that the relative birth rates of “fit” vs. “unfit” individuals in the reconstructed phylogeny are higher than in the population, and mutation rates from “unfit” to “fit” individuals are enhanced. These biases exemplify the importance of incorporating the sampling process into phylogenetic simulations.

**Figure 2:**
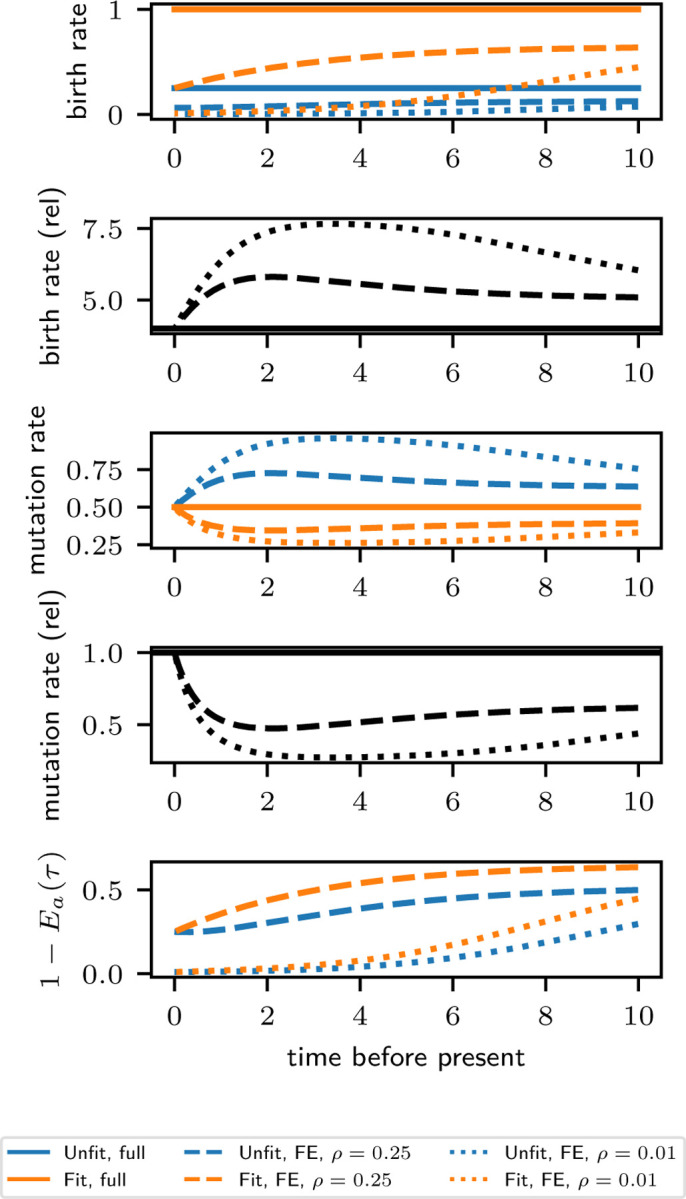
Full and forward equivalent model parameters as a function of time in a two-type model with a Fit and Unfit phenotype, with $\lambda_{\text{Fit}}=1.0$, $\lambda_{\text{Unfit}}=0.25$, $\mu_{\text{Fit}}=\mu_{\text{Unfit}}=0.25$, and anagenetic mutation rates $\gamma_{\text{Fit},\text{Unfit}}=\gamma_{\text{Unfit},\text{Fit}}=0.5$. The relative birth rate is defined as $\lambda_{\text{Fit}}\left(\tau \right)/\lambda_{\text{Unfit}}\left(\tau \right)$ and $\lambda_{\text{Fit}}^{\text{FE}}\left(\tau \right)/\lambda_{\text{Unfit}}^{\text{FE}}\left(\tau \right)$. The relative mutation rate is is defined as $\gamma_{\text{Fit},\text{Unfit}}\left(\tau \right)/\gamma_{\text{Unfit},\text{Fit}}\left(\tau \right)$ and $\gamma_{\text{Fit},\text{Unfit}}^{\text{FE}}\left(\tau \right)/\gamma_{\text{Unfit},\text{Fit}}^{\text{FE}}\left(\tau \right)$.

As a concrete example, we plot in Figure 2 the forward equivalent model parameters in a two-type model with a Fit and Unfit phenotype, with $\lambda_{\text{Fit}}=1.0$, $\lambda_{\text{Unfit}}=0.25$, $\mu_{\text{Fit}}=\mu_{\text{Unfit}}=0.25$, and anagenetic mutation rates $\gamma_{\text{Fit},\text{Unfit}}=\gamma_{\text{Unfit},\text{Fit}}=0.5$. We consider both a low sampling probability ρ=.01 and moderate sampling probability ρ=.25, occurring uniformly across phenotypes at the present. We observe that the forward equivalent birth rate is smaller than the that in the population for both the Fit and Unfit phenotypes. Moreover, we see that in the forward equivalent model, birth rates are higher in the distant past than in the present. This heightened diversification rate in the observed phylogeny in the distant past has been termed the “push of the past” [2] and results from survivorship bias. We also plot the relative birth rate in the forward equivalent model, defined as $\lambda_{\text{Fit}}^{\text{FE}}\left(\tau \right)/\lambda_{\text{Unfit}}^{\text{FE}}\left(\tau \right)$. We see that it is larger than 4, indicating that the birth rate of the Unfit phenotype is reduced more relative to its population value than that of the Fit phenotype. This effect is stronger when ρ is smaller. These qualitative phenomena can be easily seen in the formulas in (3). Indeed, the amount by which the birth rate is depressed is controlled by probability a lineage will end up in the sample $1-E_{b}\left(\tau \right)$, so that the qualitative time dependence of the birth rates, the inflation of the relative birth rate, and the dependence of the relative birth rate on *ρ* can be read off the plot of $1-E_{a}\left(\tau \right)$ in Figure 2. We also observe that the mutation rate from the Unfit to Fit phenotype is higher and from the Fit to Unfit phenotype is lower in the forward equivalent model than in the population, with a stronger effect for smaller ρ. This is the result of selective pressures, which are more pronounced when ρ is smaller. Again, (3) indicates the relevant quantity is the ratio $\left(1-E_{b}\left(\tau \right)\right)/\left(1-E_{a}\left(\tau \right)\right)$, the qualitative behavior of which can also be observed in the plot of $1-E_{a}\left(\tau \right)$ in Figure 2.

### 3.4 Efficiency improvements of forward-equivalent simulations

As we justified in Section 3.1, our simulation algorithm has time complexity which is linear in the number of simulated events. The forward-equivalent simulation can be faster than the full simulation because it simulates fewer events per non-empty reconstructed phylogeny.

There are two reasons why the forward-equivalent simulation simulates fewer events per non-empty reconstructed phylogeny. First, the reconstructed phylogeny contains fewer events (i.e., nodes) than the full phylogeny. For example, in a pure birth process that samples present-day lineages with probability ρ, the reconstructed tree will have approximately ρ times as many nodes as the full phylogeny. We thus expect the forward-equivalent simulation to run 1/ρ times faster per tree than the full simulation.

Second, in the full simulation, the reconstructed phylogeny may be empty. Thus, the full simulation may require multiple retries to produce a single non-empty reconstructed phylogeny. The events simulated in trees with empty reconstructed phylogenies contribute to the computational cost of simulating a single non-empty phylogeny. In contrast, the forward-equivalent simulation never produces the empty tree T0. As an artificial extreme case, consider a trivial process without birth or death and which samples present-day lineage with probability ρ. In this case, the full tree is a ‘stick’. The reconstructed tree is a ‘stick’ with probability ρ and is T0 otherwise. The forward equivalent simulation simulates a stick in its first try, requiring $\text{Θ}\left(1\right)$ time per non-empty reconstructed tree. The full simulation requires in expectation 1/ρ retries to obtain a non-empty reconstructed tree. In this case, the speedup provided by our method is again 1/ρ.

In what follows, we study the key quantity R (resp. $R^{\text{FE}}$), which is the expected number of simulated events required to produce a single non-empty reconstructed tree in the full (resp. forward-equivalent) simulation. For a single-type birth-death process, we prove the following result:

**Proposition 1.** Consider running a birth-death process for 1 unit of time with birth rate λ, death rate μ, and extant sampling probability ρ. Then:

1. If λ=μ, then the theoretical speedup of the forward-equivalent model is:

$$\frac{R}{R^{\text{FE}}}=\frac{1}{\rho }\left(1+\mu \right).$$

1. If λ≠μ, then the theoretical speedup of the forward-equivalent model is:

$$\frac{R}{R^{\text{FE}}}=\frac{1}{\rho }\left[1+\frac{\mu \left(e^{\lambda -\mu }-1\right)}{\left(\lambda -\mu \right)e^{\lambda -\mu }}\right].$$

We prove Proposition 1 in Appendix C. In the simpler case that the birth and death rates are equal, we see that the theoretical speedup is essentially linear in the (inverse) sampling probability ρ and in the death rate μ. Later, in our empirical benchmarks, we will see that this theoretical estimate is quite accurate.

For the general BDMS model, we have the following result.

**Proposition 2.** Let T denote the full tree in the full model, and $r\left(T\right)$ its corresponding (possibly empty) reconstructed tree. Then

$$\frac{R}{R^{\text{FE}}}=\frac{E\left[\#nodes\left(T\right)\right]}{E\left[\#nodes\left(r\left(T\right)\right)\right]}$$

We emphasize here that nodes in T include birth events, mutation events, and sampling events and thus need not all be locations of bifurcation in the tree. We prove Proposition 2 in Appendix C. The significance of Proposition 2 is that it unifies the two sources of speed-up described above, avoiding explicit accounting of the number of retries required to observe a non-empty reconstructed phylogeny.

We remark that the expected number of nodes appearing in Proposition 2 can be computed numerically with time discretization and dynamic programming. Thus, Proposition 2 allows for numerical estimates of speed-up factors. Empirically, the forward-equivalent simulation may experience a constant factor computational overhead relative to the full simulation due to its sampling from time inhomogeneous Poisson processes: see Section 4.2. In our experiments, this overhead was smaller than a factor of 2, so the forward-equivalent simulation was faster than the full simulation for moderate values of ρ and μ.

## 4 Empirical validation

We carry out several simulations to validate our method. In Section 4.1, we consider a case where both the full and forward-equivalent simulations are feasible, and perform several tests of whether the reconstructed phylogenies produced by the two simulations have the same distribution. In Section 4.2, we perform several simulations to measure the relative computational efficiency of the two approaches and to validate the theory from Section 3.4. In Section 4.3, we use the forward-equivalent simulation to generate phylogenies from a setting in which the full simulation is computationally infeasible on our machines.

### 4.1 Distribution tests

We performed a simulation study to test that the full and forward-equivalent simulations produce reconstructed phylogenies with the same distribution. We consider a full model consisting of two types, Fit and Unfit. The population is simulated for $t_{\max}=20$ units of biological time. Both types have a death rate of $\mu_{\text{Fit}}=\mu_{\text{Unfit}}=0.25$, the type Unfit has birth rate of $\lambda_{\text{Unfit}}=0.25$, and the type Fit has a birth rate of $\lambda_{\text{Fit}}=1$. Recall that, without mutation and given infinite time, a population will go extinct with probability 1 if and only if the birth rate does not exceed the death rate: λ≤μ. Thus, we have chosen parameters so that the Unfit population is just barely in the regime where it would, without mutation, be assured eventual extinction. On the other hand, the Fit population would, without mutation, be unlikely to go extinct and would grow exponentially fast. We consider anagenetic mutation rates $\gamma_{\text{Fit},\text{Unfit}}=0.8$ and $\gamma_{\text{Unfit},\text{Fit}}=0.1$. Thus, deleterious mutations from Fit to Unfit are much more common than beneficial mutations from Unfit to Fit. Based on our simulations (see below), these mutation rates keep the distribution of types in the population relatively balanced. We consider a model with sampling at the present $\rho_{\text{Fit},0}=\rho_{\text{Unfit},0}=0.5$ and no sampling at earlier times or at death events.

We generated 1000 non-empty phylogenies from both the full and forward-equivalent simulations for the above model. Because we are conditioning on non-emptiness, generating a single non-empty phylogeny in the full simulation may (and sometimes did) require multiple retries.

We tested distributional equivalence of several scalar summary tree statistics. Because phylogenetic trees are high-dimensional objects, distributional agreement on these test statistics does not necessarily imply distributional equivalence of the phylogenetic trees. Moreover, the tree statistics we selected are not intended to be exhaustive, and we do not intend to imply that they provide an optimal summary of tree geometry for any particular application. Rather, we intend to capture a wide enough range of tree features to provide convincing evidence that the distributional equivalence (1) holds. The tree statistics we considered were:

- **Event count.** The total number of nodes in the tree, which includes births, mutations, and sampled leaf nodes.
- **Leaf count.** The total number of leaves in the tree. In this simulation, this is the size of the sample from the present-day population.
- **Branch length.** The cumulative branch length stratified by type (for Fit and Unfit lineages) and in total.
- **Subtree count, stratified by size.** The number of nodes in the tree for which the descendant tree has size nodes (including the root node). For example, the sub-tree count at size = 1 is the number of leaves. The sub-tree count at size = 3 contains the sum of the number of cherries and the number of nodes after which one mutation event but no birth events occur before present-day sampling.
- **Lineage count, stratified by type and time.** For a type in {Fit, Unfit} and time t, the number of lineages in the tree of that type at a cross-section at time t.

We also considered the distribution of “block statistics”. In multi-type models, each phylogeny is partitioned into a collection of maximally connected sub-trees of uniform type. We call each element of this partition a *block*. A block statistic is a scalar summary of a block; for example, total branch length. We simulated 1000 non-empty trees and then plot histograms of various block statistics over the list of generated blocks. We emphasize that not all trees contain the same number of blocks, so that trees with more blocks contribute more counts to these histograms than trees with fewer blocks. The histograms represents a distribution which assigns an equal weight to each block, and thus assigns a weight to each tree proportional to its number of blocks. We consider the following block statistics:

- **Events per block, stratified by type.** The number of nodes in the block.
- **Branch length per block, stratified by type.** The cumulative branch length of the block.

**Figure 3:**
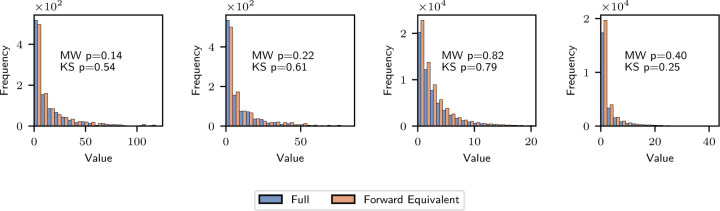
Distributional comparison of summary statistics from two-type model described in the text. The full and forward equivalent simulation are both run until 1000 non-empty trees are generated. Each plot displays a different summary statistics and the Mann-Whitney (MW) and Kolmogorov-Smirnov (KS) p-value testing the equivalence of the full and forward equivalent simulations. Left: number of extant lineages at biological time t=16 with phenotype Unfit. Middle-left: number of extant lineages at biological time t=16 with phenotype Fit. Middle-right: branch length of blocks with phenotype Unfit. Right: branch length of blocks with phenotype Fit.

In Figure 3, we show histograms for two of these summary statistics—lineage count and branch length per block—stratified by type. For the block-based statistics (in this case, branch length per block), the *y*-axis counts the number of blocks cumulatively across phylogenies, so that each phylogeny may contribute multiple blocks to the count, and larger phylogenies will typically contribute more. In all cases, the histograms generated by the full and forward-equivalent simulations are visually similar. For each summary statistic, we performed a two-sided Mann-Whitney U-test and the Kolmogorov-Smirnov test to compare the distributions, with the *p*-values in each case reported on the corresponding histogram plot. We caution that the theoretical null distribution used to compute Kolmogov-Smirnov test statistic is only asymptotically valid for continuous distributions. Thus, it must be interpreted with caution for the count-based test statistics when the counts are small. Similar plots to Figure 3, reporting results for all the summary statistics listed above, are provided in Supplementary Figure 6.

While not a proof of distributional equivalence, these tests indicate that our implementation successfully produces the distributional equivalence predicted by Theorem 1, providing evidence for its correctness.

### 4.2 Computational efficiency

We empirically assessed the relative computational cost of the full and the forward-equivalent simulations and their dependence on population size and the size of the sub-sampled phylogeny under three models: single-type without death, single-type with death, and two-type.

The “single-type without death” model consisted of a unique type with constant birth rate given by λ=1.0 and no death run for $t_{\max}=5$ units of biological time. We subsampled the present population with probability ρ, which we set to ρ=0.1×k for k=1,…,10. At each value of k, we simulated 50 non-empty trees. As in Section 4.1, the full simulation may require multiple retries to generate a single non-empty tree.

**Figure 4:**
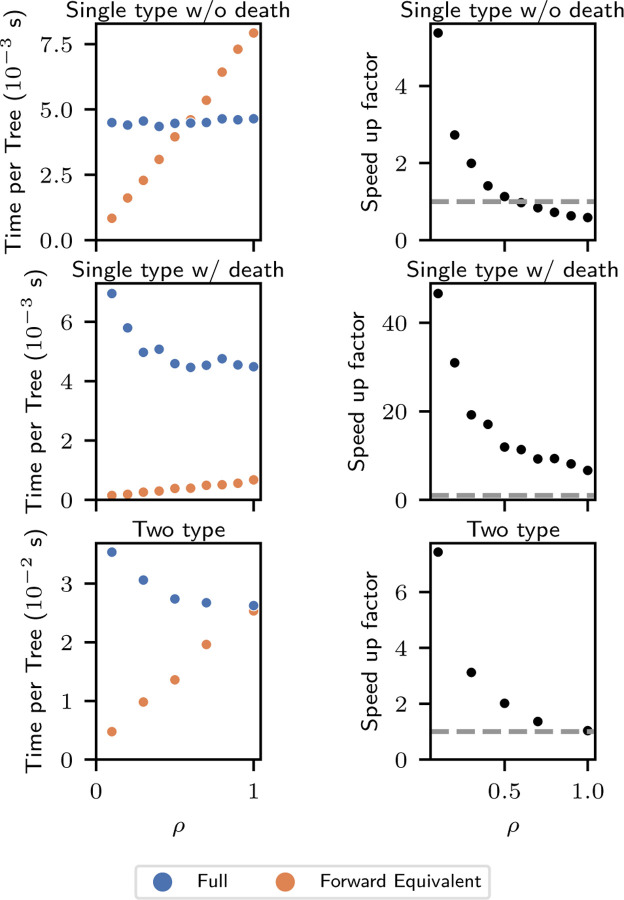
Comparison of computational costs. Time per tree (s) is the total amount of simulation time required to produce 50 non-empty trees, divided by 50. Speed up factor is the ratio of the time per tree from the full simulation to the forward equivalent simulation. All sampling occurs at present with probability ρ for each extant lineage identically across types. Horizontal dashed line at speed up factor = 1. A: single-type without death. λ=1.0, μ=0, $t_{\max}=5$. B: single-type with death. λ=1.0, μ=1.0, $t_{\max}=10$. C: two-type with death. Two types: Fit and Unfit. $\lambda_{\text{Fit}}=1.0$, $\lambda_{\text{Unfit}}=0.25$, $\mu_{\text{Fit}}=\mu_{\text{Unfit}}=0.25$.

All results are displayed in Figure 4. In the left column we report, for each value of ρ, the average total simulation time (including retries) required to produce 50 non-empty trees, normalized by 50. In the right column, we report the ratio of the computation time required by the full simulation to the forward-equivalent simulation on a log-log scale.

The first row shows results for the single-type model without death. For the full simulation, the mean time per tree is approximately constant in ρ. For the forward-equivalent simulation, the mean time per tree appears to be linear in ρ. This is consistent with our simulation scaling linearly in the total number of simulated events, which in the full simulation is independent of ρ and in the forward-equivalent simulation is proportional to ρ on average.

The speed-up factor is decreasing with ρ, and is larger than 1 for ρ≤0.5, reaching ≈ 5 at ρ=0.1. Regressing the log speed up factor on logρ gives the approximation speed up $\text{factor}\approx 0.58\rho^{-0.99}$, consistent with the inverse dependence on ρ predicted by Proposition 1. The speed-up factor is smaller by approximately a factor of 2 relative to the theory. We believe this is because, in this model, the forward-equivalent incurs some overhead relative to the full simulation because it must sample from time-inhomogenous rather than constant-rate Poisson processes (see Appendix A for implementation details). For ρ≳0.6, the gain from avoiding simulating unobserved events does not overcome this computational overhead, and the speed-up factor is less than one. We emphasize, however, that this computational overhead increases computation time by a constant factor which does not grow with phylogeny size. Thus, it will be overcome by a small amount of subsampling even for very large phylogenies. For models that do not include death, the forward-equivalent simulation is faster than the full simulation when there is at least a moderate amount of subsampling. When the full model itself includes time-inhomogeneous rate parameters, the forward-equivalent simulation will not have this computational overhead relative to the full simulation.

In the second row of Figure 4, we present similar results for a single-type model that includes death. Precisely, we consider a regime with birth rate λ=1.0 and death rate μ=1.0, run for $t_{\max}=10$ units of biological time. The computational savings are even more dramatic in this case, at ρ=0.1 reaching a speed-up factor of ≈ 45 and at ρ=1 a speed-up factor of ≈ 6. In this case, even at ρ=1, the forward-equivalent simulation is much faster than the full simulation. This is because lineages that die are not sampled. For models that include death, the forward-equivalent simulation can be more efficient for all sub-sampling schemes.

We also observe that the computation time for the full simulation is no longer approximately constant in the sampling probability ρ. In fact, unlike in the forward-equivalent simulation, the computation tends to be larger for smaller values of ρ. We suspect this is a result of the computational cost of pruning the phylogeny, which is more computationally intensive when a smaller fraction of the tree is sampled.

Finally, in the third row of Figure 4, we present similar results for the two-type model described in Section 4.1, and we observe similar phenomena to that appearing in the single-type simulations.

In Supplementary Figure 2, we present further plots demonstrating the computational overhead described above and the linear scaling of computation time in the total number of simulated events.

### 4.3 Subsampling from massive phylogenies

The previous section focused on situations in which, although the forward-equivalent simulation was much faster than the full simulation, the phylogeny sizes were small enough that both approaches were computationally feasible. The computational cost of the forward-equivalent simulation scales with the size of the sub-sampled rather than the full phylogeny. Thus, in principle, it allows for simulation of phylogenies subsampled from arbitrarily large populations.

As an example, we consider a multi-type model with two types Unfit and Fit with birth rates $\lambda_{\text{Unfit}}=0.25$ and $\lambda_{\text{Fit}}=0.25$, death rates $\mu_{\text{Unfit}}=0.25$ and $\mu_{\text{Fit}}=0.5$, and mutation rates $\gamma_{\text{Unfit},\text{Fit}}=0.1$ and $\gamma_{\text{Fit},\text{Unfit}}=0.25$, respectively. Thus, the Fit type has higher birth rates and death rates but a larger net speciation rate $\lambda_{\text{Fit}}-\mu_{\text{Fit}}>\lambda_{\text{Unfit}}-\mu_{\text{Unfit}}$ than the Unfit type. Moreover, deleterious mutations occur at a higher rate than beneficial mutations. We simulate the population for biological time $t_{\max}=47$ and sample each lineage in the present population with probability $\rho =10^{-9}$. With these simulation parameters, we find we often generate trees with leaf counts in the 10s to 100s. This indicates that the full population size from which the phylogeny is sampled is in the 10s to 100s of billions. On a Macbook Pro with an Apple M1 Pro chip, each tree in this simulation required just over a second of computation time on average.

**Figure 5:**
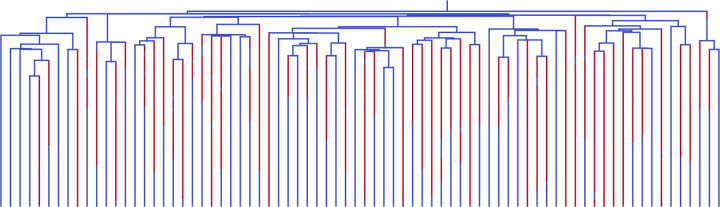
A sub-sampled phylogeny from a multi-type model with population size in the billions. The Fit type is in blue and the Unfit type is in red.

In Figure 5, we display a typical phylogeny drawn from this simulation. From such a tree, we see several features that are relevant to understanding phylogenies generated from experiments involving billions of cells. In particular, we observe that most birth events occur well before the present, and most (but not all) mutations occur after the most recent observed birth event. Most lineages mutated to the Unfit type shortly after the most recent birth event, but mutated again to the Fit type before sampling at the present day. Further information regarding observed mutation rates and birth rates, their time dependence, and the behavior of various approaches to estimating underlying process parameters, can also be investigated empirically using such simulated phylogenies.

## 5 Discussion

This paper has proposed a novel method for simulating reconstructed phylogenies that leads to substantial computational savings when there is sparse sampling or high levels of death in the population. Many scientific studies rely on extensive phylogenetic simulation. Unfortunately, when the full population size is in the billions or more, as is typical in epidemiological, cancer evolution, and antibody evolution applications, the cost of the full simulation is prohibitive. The forward-equivalent simulation we propose makes simulating the reconstructed phylogeny feasible in these domains when the set of sampled lineages is substantially smaller than the full population, enabling much more realistic simulations and benchmark studies.

We believe that the forward-equivalent simulation approach will facilitate improved inference of population dynamics. We describe some directions for future work.

- **Training of machine learning models.** Recently, deep learning approaches for estimating epidemiological parameters from reconstructed phylogenies have been proposed [31]. These methods are often trained on large datasets of phylogenies simulated with different model parameters. By enabling faster simulation, our method can enable the training of such models. In particular, the forward-equivalent simulation can allow the simulated phylogenies to be faithful to realistic population sizes common in epidemiological, cancer evolution, and other domains.
- **Simulation at inference-time.** Some inferential methods require simulation at inference time. For example, “Approximate Bayesian Computation” involves computing summary statistics of observed phylogenies, and then stochastically searching for model parameters which, in simulation, generate phylogenies with similar summary statistics [33]. A computational bottleneck in these approaches is the cost of simulation. We anticipate the forward-equivalent simulation may improve or enable simulation-based approaches to inference.
- **Identifiability.** Following the seminal paper of Louca and Pennell (*Nature*, 2021), there has been substantial interest in issues of identifiability in birth-death models [12–14]. These papers study single-type models with time-varying birth and death rates, and show that, due to sub-sampling, there are multiple values of the underlying process parameters that can induce the same distribution over observed phylogenies. This calls into question the ability to use phylogenies to reconstruct parameters driving diversification and extinction dynamics in the past. Our simulation approach is related to a lack of identifiability in multi-type models: indeed, the full model and the forward-equivalent model induce the same distribution over reconstructed phylogenies, so cannot be distinguished using the observed data alone. Thus, this paper establishes that the identifiability issues that occur in single-type models also occur in more general multi-type models, and we specify for each full model a different model that belongs to the same equivalence class. The current paper identifies the utility of this lack of identifiability for the purpose of simulation, but it will also be important to investigate the challenges this introduces for the purpose of inference. Moreover, a complete description of the classes of indistinguishable models and investigation of assumptions that may resolve identifiability issues, as in [12, 13], is left to future work.

## 6 Materials and methods

### Software implementation.

We have made reproducible analysis available on GitHub.^2^ We used the Python package BDMS [3]—which is developed by us and other collaborators—as a reference implementation of the full simulation, and developed wrapper code to implement the forward-equivalent mapping $\text{Θ}\mapsto {\text{Θ}}^{\text{FE}}$and perform simulation studies. We use the Python package ETE [9] for tree manipulation and visualization.

## Supplementary Material

Supplement 1

## Figures and Tables

**Table 1:** Summary of processes in the birth-death-mutation-sampling (BDMS) model.

Process	Description
**Initialization**	At time $t_{\max}$, the population begins with one lineage with type a~π.
**Birth**	Type a splits into types a and b at rate $\lambda_{a,b}\left(\tau \right)$ at time τ.
**Death**	Type a dies at rate $\mu_{a}\left(\tau \right)$ at time τ.
**Anagenetic mutation**	Type a mutates to type b at rate $\gamma_{a,b}\left(\tau \right)$ at time τ.
**Sampling**	Type τ gets sampled at rate $\psi_{a}\left(\tau \right)$ at time τ. If sampled, it dies with probability $r_{a}\left(\tau \right)$ at time τ.
**CSEs**	At time $t_{l}$, type a gets sampled with probability $\rho_{a,l}$. If sampled, it dies with probability $r_{a,l}$.

## Footnotes

1 We make no distinction between phylogenetic and genealogical trees in this work.

2 Code available at https://github.com/songlab-cal/forward-equivalent-trees
