# Supplementary material for: Exact and efficient phylodynamic simulation from arbitrarily large populations
Source: ArXiv. 2024 Aug 10:arXiv:2402.17153v2. Originally published 2024 Feb 27. Preprint. [Version 2] (PMC10925381)
Supplement: 1 [file NIHPP2402.17153V2-supplement-1.pdf]

Supplementary Figures

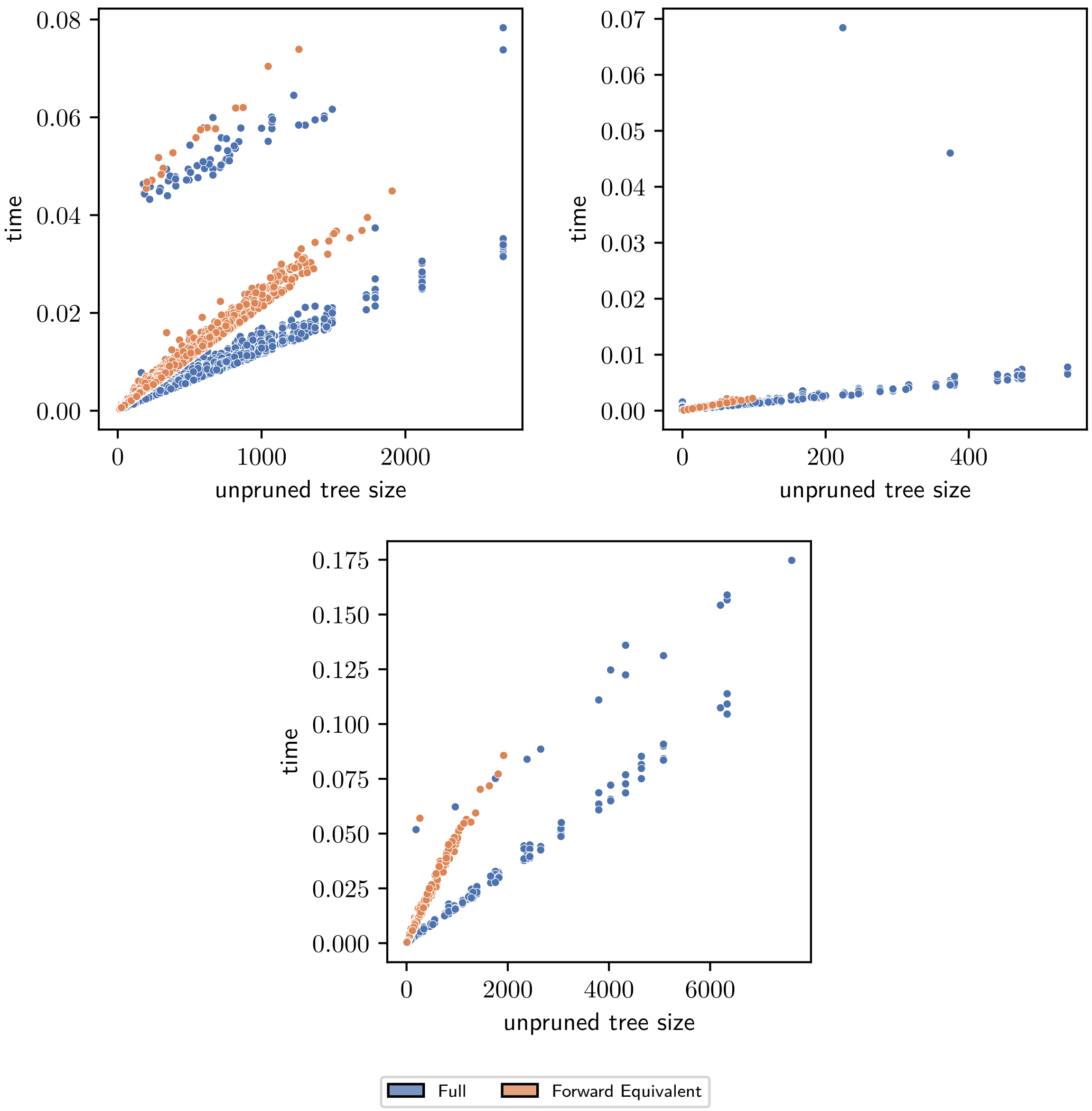

Figure S1: Computation time required for each individual tree against the unpruned tree size across various simulation settings. We see that, in correspondence with the discussion in Appendix A and Section 3.4, the computation time is approximately linear in the size of the unpruned tree. The forward-equivalent model is slower than the full simulation after controlling for the size of the unpruned tree due to the computational overhead of simulating from a BDMS model with time-varying rates. The greater efficiency of the forward-equivalent simulation comes from simulating trees with substantially smaller unpruned tree size. Top left: single-type model without death. Top right: single-type model with death. Bottom: multi-type model. Each point is a single tree.

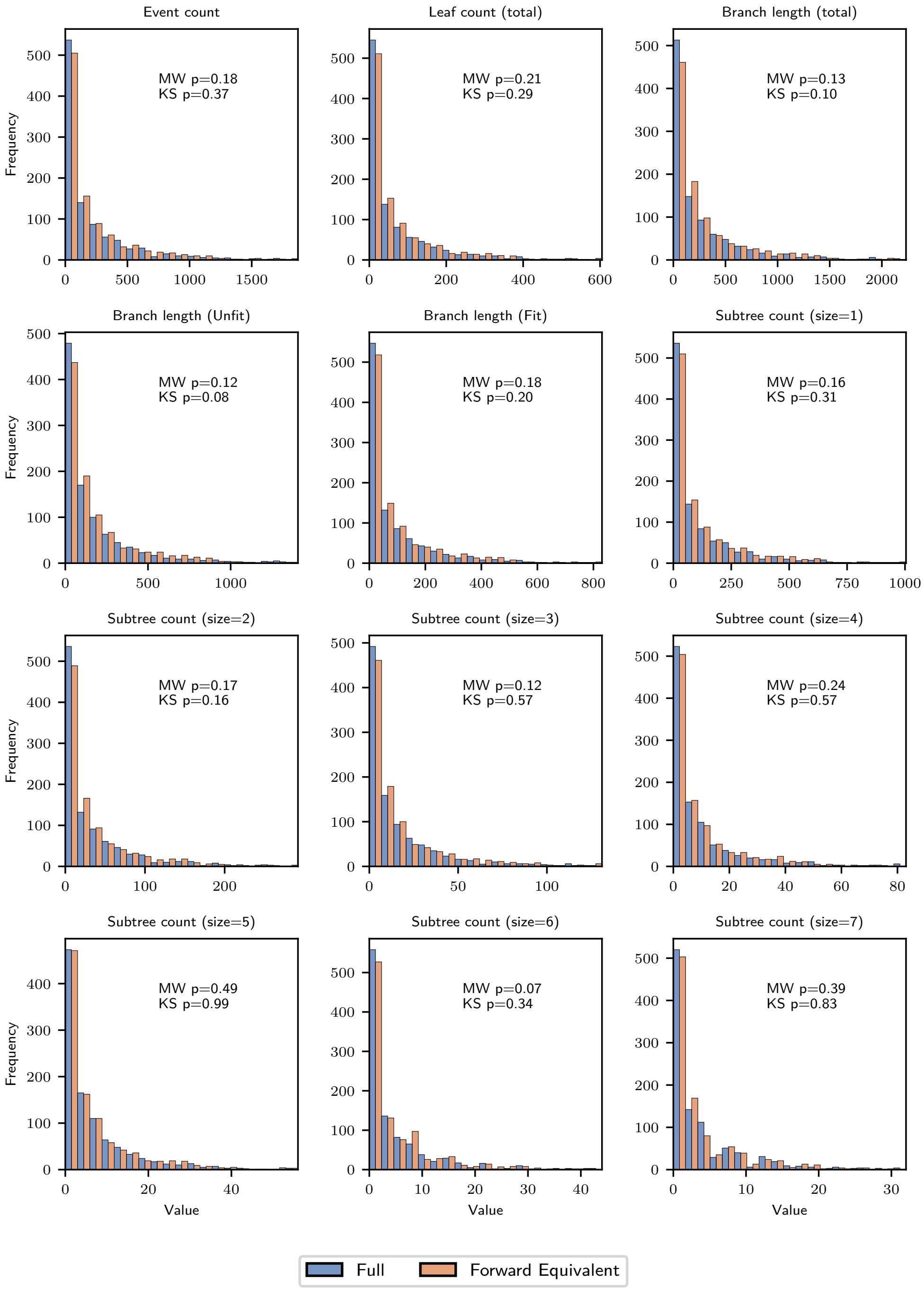

Figure S2: Distributional comparisons analogous to those appearing in Figure 3, for all summary statistics we considered, as described in Section 4.1. We emphasize that because all of these tests were computed using the same sample of phylogenies, the p-values for different tests need not be independent.

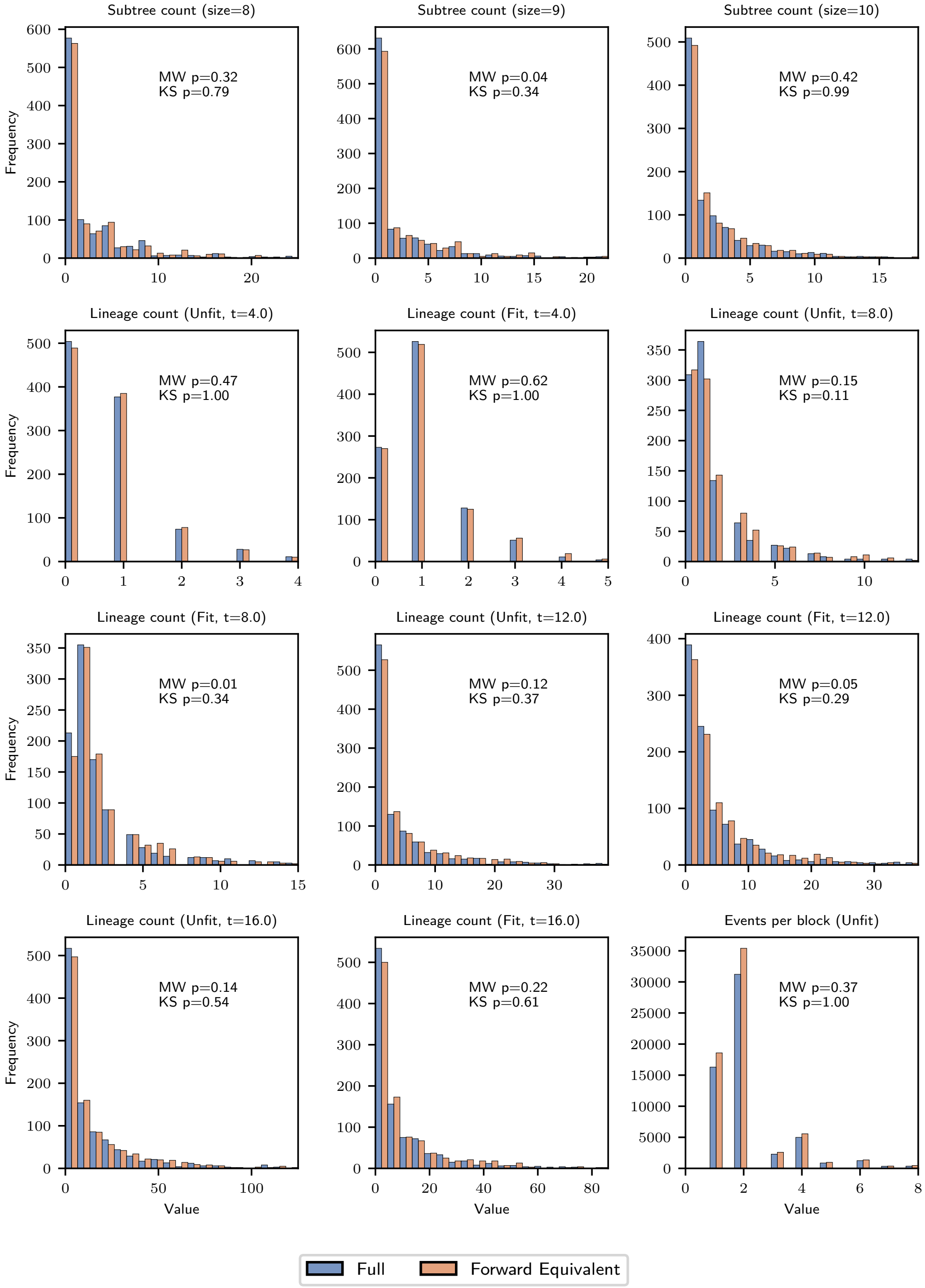

Figure S2: (Continued)

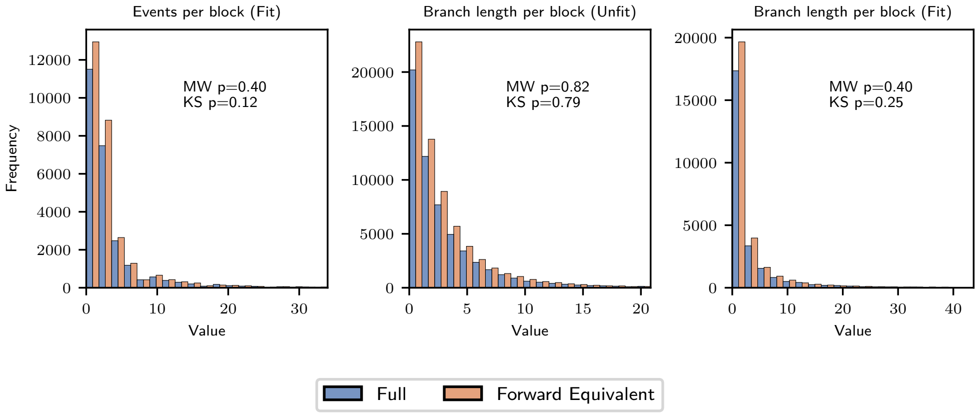

Figure S2: (Continued)
