# Supplementary material for: Exact and efficient phylodynamic simulation from arbitrarily large populations
Source: ArXiv. 2024 Feb 27:arXiv:2402.17153v1. Preprint. [Version 1] (PMC10925381)
Supplement: Supplementary file 1 [file NIHPP2402.17153v1-supplement-1.pdf]

Supplementary Information

A Further implementation details

In this section, we describe additional implementation details to enable Algorithm 1 to run in time which scales linearly in the number of events that occur in the full population. First, we must implement GetNextEvent in constant time in the population size, which is described in Section A.1. We must also store, at any given time, the active population in a data structure that supports insertion, deletion, and selection of an element uniformly at random, in constant time in the population size, which is described in Section A.2. Finally, our ability to implement GetNextEvent requires we perform precomputations before simulation time. These are described in Section A.3.

A.1 Implementing GetNextEvent in constant time in the population size

As described in Section 3.1, in order for Algorithm 1 to have time complexity linear in the number of events in the full phylogeny, we must implement the function GetNextEvent in constant time in the population size. Our implementation relies on the following fact. Consider a Poisson point process with time-varying rate given by $\theta(\tau) \geq 0$. Let

$$\Theta(\tau, \tau') := \int_{\tau}^{\tau'} \theta(\tau)\mathrm{d}\tau, \qquad \Theta^{-1}(\tau, z) := \sup\{\tau' \in \mathbb{R} \mid \Theta(\tau, \tau') \leq z\}. \tag{4}$$

Consider n iid copies of this Poisson point process. For a fixed time τ, consider the next time after τ that an event occurs in any one of these n processes. It is routine to show that this arrival time has distribution given by $\Theta^{-1}(\tau, Z/n)$ where $Z \sim \mathrm{Exp}(1)$ has exponential distribution with rate parameter 1 (that is, has density given by e^{-z} for $z \geq 0$). Note that $\Theta(\tau, Z/n)$ can be infinite if $\lim_{\tau' \to \infty} \Theta(\tau, \tau') < \infty$ and $Z/n \geq \lim_{\tau' \to \infty} \Theta(\tau, \tau')$. This corresponds to the event that none of the n Poisson processes have any events occur after time τ, which can occur when the Poisson rate function is integrable.

The GetNextEvent function takes in the current time τ and the number of extant lineages of each type $|S_a|$. Given the discussion in the previous paragraph, this provides a way of determining when the next event in the population occurs, and what type it is, where the type specifies whether it is a birth, death, mutation, or sampling event, and the types of the parent and child nodes in the phylogeny. For each event type, we generate the time of the next event of that type. The next event type is chosen to be the one that happens the soonest among these generated times. Formally, for $a, b = 1, \ldots, d$, define

$$\begin{aligned} \Lambda_{a,b}(\tau_1, \tau_2) &= \int_{\tau_1}^{\tau_2} \lambda_{a,b}(\tau)\mathrm{d}\tau, \qquad & M_a(\tau_1, \tau_2) &= \int_{\tau_1}^{\tau_2} \mu_a(\tau)\mathrm{d}\tau, \\ \Gamma_{a,b}(\tau_1, \tau_2) &= \int_{\tau_1}^{\tau_2} \gamma_{a,b}(\tau)\mathrm{d}\tau, \qquad & \Psi_a(\tau_1, \tau_2) &= \int_{\tau_1}^{\tau_2} \psi_a(\tau)\mathrm{d}\tau. \end{aligned} \tag{5}$$

Then define

$$\begin{aligned} \Lambda_{a,b}^{-1}(\tau, z) &= \sup\{\tau' \in \mathbb{R} \mid \Lambda_{a,b}(\tau, \tau') \leq z\}, \qquad & M_a^{-1}(\tau_1, z) &= \sup\{\tau' \in \mathbb{R} \mid M_a(\tau, \tau') \leq z\} \\ \Gamma_{a,b}^{-1}(\tau_1, z) &= \sup\{\tau' \in \mathbb{R} \mid \Gamma_{a,b}(\tau, \tau') \leq z\} \qquad & \Psi_a^{-1}(\tau_1, z) &= \sup\{\tau' \in \mathbb{R} \mid \Psi_a(\tau, \tau') \leq z\}, \end{aligned} \tag{6}$$

where these can be possibly infinite (for example, if one of the rate parameters is 0). We draw exponential random variables $\{Z_{a,b}^{\gamma}\}_{a,b=1}^{d}$, $\{Z_a^{\mu}\}_{a=1}^{d}$, $\{Z_{a,b}^{\gamma}\}_{a,b=1}^{d}$, $\{Z_a^{\psi}\}_{a=1}^{d}$ iid from Exp(1) so that

we have one exponential random variable for each event type. The occurrence of the next birth event $a \to a, b$ occurs at time $\Lambda_{a,b}^{-1}(\tau, Z_{a,b}^{\lambda}/|S_a|)$; the next death of a lineage with type a occurs at time $M_a^{-1}(\tau, Z_a^{\mu}/|S_a|)$; the next anagenetic mutation $a \to b$ occurs at time $\Gamma_{a,b}^{-1}(\tau, Z_{a,b}^{\gamma}/|S_a|)$; and the next sampling event occurs at $\Psi_a^{-1}(\tau, Z_a^{\psi}/|S_a|)$. The next event of any type occurs at the minimum of these, and the type of the next event is determined by which process achieves the minimum. Thus, GetNextEvent can be implemented by the pseudo-code in Algorithm 3. Provided $\Lambda_{a,b}^{-1}(\tau, Z_{a,b}^{\lambda}/|S_a|)$, $M_a^{-1}(\tau, Z_a^{\mu}/|S_a|)$, $\Gamma_{a,b}^{-1}(\tau, Z_{a,b}^{\gamma}/|S_a|)$, $\Psi_a^{-1}(\tau, Z_a^{\psi}/|S_a|)$ can be evaluated

Algorithm 3 GetNextEvent($\{|S_a|\}, \tau$)

Draw $\{Z_{a,b}^{\gamma}\}_{a,b=1}^{d}$, $\{Z_a^{\mu}\}_{a=1}^{d}$, $\{Z_{a,b}^{\gamma}\}_{a,b=1}^{d}$, $\{Z_a^{\psi}\}_{a=1}^{d}$ iid from Exp(1);
$\tau_* \leftarrow \min_{a,b} \left\{\Lambda_{a,b}^{-1}(\tau, Z_{a,b}^{\lambda}/|S_a|), M_a^{-1}(\tau, Z_a^{\mu}/|S_a|), \Gamma_{a,b}^{-1}(\tau, Z_{a,b}^{\gamma}/|S_a|), \Psi_a^{-1}(\tau, Z_a^{\psi}/|S_a|)\right\}$;
$\text{EventType} \leftarrow \arg\min \left\{\Lambda_{a,b}^{-1}(\tau, Z_{a,b}^{\lambda}/|S_a|), M_a^{-1}(\tau, Z_a^{\mu}/|S_a|), \Gamma_{a,b}^{-1}(\tau, Z_{a,b}^{\gamma}/|S_a|), \Psi_a^{-1}(\tau, Z_a^{\psi}/|S_a|)\right\}$;
if $\tau^* < 0$ **then return** $(0, \text{EventType})$;
else return $(\tau^*, \text{EventType})$;

in constant time, the time complexity of Algorithm 3 is $O(d^2)$, and in particular, does not grow with population size.

In the case that the time of the next event occurs after the present day, we instead exit the loop and advance all extant lineages to the present day. We then carry out the present day sampling process: each lineage is sampled with probability $\rho_{a,0}$, and upon sampling, goes extinct with probability $r_{a,0}$. If our model includes CSEs prior to the present day, we follow a similar logic when the next event time occurs after the next CSE. We can thus implement GetNextEvent using the pseudo-code in Algorithm 3 complemented by the additional CSE logic. For simplicity, the pseudo-code represents a case without CSEs.

In some cases (for example, for constant rate Poisson processes), $\Gamma_{a,b}^{-1}$, M_a^{-1}, $\Lambda_{a,b}^{-1}$, and Ψ_a^{-1} have analytic forms that permit constant time evaluation. When analytic forms are not available (which is typically the case for the forward-equivalent models), we leverage precomputations. Note that $\Theta^{-1}(\tau, z) = \Theta^{-1}(0, \Theta(0, \tau) + z)$. Thus, to allow for constant time evaluation of Θ^{-1} at simulation time, we can precompute Θ at an equispaced grid of $\tau \in [0, t_{\max}]$ and Θ^{-1} on an equispaced grid of $z \in [0, \Theta(0, t_{\max})]$. We describe the precomputations for $\Lambda_{a,b}^{-1}$, $M_a^{-1}(0, z)$, $\Gamma_{a,b}^{-1}(0, z)$, $\Psi_a^{-1}(0, z)$ in Section A.3. Then, at simulation time, we compute $\Theta(0, \tau)$ and $\Theta^{-1}(0, \Theta(0, \tau) + z)$ by linear interpolation with respect to this mesh. This is a constant time operation, because, for example, the interval in which τ occurs has left and right indices (using Python indexing) given by $\lfloor \tau/\Delta\tau \rfloor$ and $\lceil \tau/\Delta\tau \rceil$, where $\Delta\tau$ is the size of the gaps in the discrete grid. Thus, the evaluation requires index-based array lookup.

We remark that in our code (see Section 6), the implementation of the mutation process is slightly different. The event type specifies only the type of the type a experiencing the mutation, not the type b into which it mutates. Conditional on type a mutating, we draw type b from the appropriate distribution. In particular, rather than compute $\Gamma_{a,b}(\tau_1, \tau_2)$, we compute

$$\Gamma_a(\tau_1, \tau_2) = \int_{\tau_1}^{\tau_2} \sum_b \gamma_{a,b}(\tau) \mathrm{d}\tau, \tag{7}$$

and define $\Gamma_a^{-1}(\tau, z)$ in the obvious way. Then, conditional on a mutation occurring to type a at time τ^*, we draw b from $\mathbb{P}(b) = \gamma_{a,b}(\tau^*)/\sum_{b'} \gamma_{a,b'}(\tau^*)$. We do this based on the API of the BDMS simulation engine we use [3], but do not claim there is any intrinsic advantage or disadvantage of either approach relative to the other.

A.2 Randomized Set

Algorithm 1 requires keeping sets of pointers to the active nodes in the population of each type at each point in time τ. This data structure must support insertion and deletion (see, for example, line 11 of Algorithm 1) as well as the ability to drawn an element of uniformly at random (see, for example, line 16 of Algorithm 3). Such a data structure is called a "randomized set," and is the data structure we use in our code (see Section 6).

A.3 Precomputation

As described in Section A.1, in the absence of analytic forms, we compute $\Theta^{-1}(\tau, z)$ by precomputing $\Theta(0, \tau)$ and $\Theta^{-1}(0, z)$ on a discrete grid, for Θ corresponding to $\Gamma_{a,b}$, M_a, $\Lambda_{a,b}$, or Ψ_a. We here describe this precomputation for the forward-equivalent model.

For each type a, we computed $E_a(\tau)$ according to (2) using Euler's method on $[0, t_{\max}]$ with time discretization $\Delta\tau = .01$. This gives $E_a(\tau)$ on a discrete grid, which we extend to any τ by linear interpolation. The model parameters $(\pi^{\mathsf{FE}}, \lambda^{\mathsf{FE}}_{a,b}, \mu^{\mathsf{FE}}_a, \psi_a, r^{\mathsf{FE}}_a, \gamma^{\mathsf{FE}}_{a,b}, \rho^{\mathsf{FE}}_{a,l}, r^{\mathsf{FE}}_{a,l})$ can then be precomputed on the same discrete grid using (3), consuming space $O(d^2 t_{\max}/\Delta\tau)$ and requiring time $O(d^2 t_{\max}/\Delta\tau)$. We then precompute the integrals $\Gamma_{a,b}(0, \tau)$, $M_a(0, \tau)$, $\Lambda_{a,b}(0, \tau)$, $\Psi_a(0, \tau)$ on the same equispaced grid using numerical integration. For example, this gives us $\Lambda_{a,b}(0, k\Delta\tau)$ for some finite sequence of integers $k = 0, 1, \ldots K$. This allows us to evaluate $\Lambda^{-1}_{a,b}(0, z)$ on the mesh $\{0, \Lambda_{a,b}(0, \Delta\tau), \ldots, \Lambda_{a,b}(0, K\Delta\tau)\}$. This mesh is not equispaced. We get an evaluation of $\Lambda^{-1}_{a,b}(0, z)$ on an equispaced grid of $[0, \Lambda_{a,b}(0, K\Delta\tau)]$ by linear interpolation of the values computed on the mesh $\{0, \Lambda_{a,b}(0, 1\Delta\tau), \ldots, \Lambda_{a,b}(0, K\Delta\tau)\}$. At simulation time, all evaluations are done using linear interpolation on these grids, as described in Section A.1.

This approach requires storing precomputations in arrays whose memory footprint is $O(d^2 t_{\max}/\Delta\tau)$. The time-complexity of the precomputation is dominated by the numerical integration of (2), whose time complexity using Euler's method is $O(d^2 t_{\max}/\Delta\tau)$. The precomputation is performed only once, so this cost is amortized over the number of simulations carried out. For the simulations in this paper, the precomputations occurred in a few microseconds. For very large type spaces (i.e., large d), it is possible that both the space and time complexity of these precomputations would become prohibitive.

A.4 Implementation based on a priority queue

An alternative simulation approach to that provided by Algorithm 1 has runtime $O(\#\text{nodes}(\mathcal{T})(d + \log(\#\text{nodes}(\mathcal{T}))))$. Thus, it may be faster when the type space is large. We briefly outline this approach here.

We again simulate forward in time τ and maintain the population of extant individuals at time τ. Additionally, we maintain a priority queue containing the next event for each individual in the extant population. Thus, the next event in the population is given by the event in the priority queue with smallest time, which can be obtained in logarithmic time in the population size by virtue of the priority queue data structure. The algorithm thus proceeds by advancing time to this event, and then performing the event. When this event creates or modifies individuals (such as upon a birth event or a mutation event), the updated or newly created individuals are added to the population of extant individuals, and their next events are immediately sampled and pushed into the priority queue.

Inserting and popping from a priority queue has time complexity which grows logarithmically with the number of elements in the priority queue, which in this case corresponds to the extant

population size. For any fixed individual, generating the next event occurring to this individual has time complexity $O(|\mathcal{A}|)$. This is due to generating the time and type of the next mutation event, which involves generating a random type from a state space of $|\mathcal{A}|$ types with non-uniform probabilities stored in some precomputed arrays. Thus, each iteration has time complexity $O(|\mathcal{A}|+\log(\#\text{nodes}(\mathcal{T})))$, and the number of iterations is equivalent to the number of events in the full phylogeny. The total time complexity is thus $O(\#\text{nodes}(\mathcal{T})(|\mathcal{A}|+\log(\#\text{nodes}(\mathcal{T}))))$. This should be compared with the time complexity $O(\#\text{nodes}(\mathcal{T})|\mathcal{A}|^2)$ of the implementation in Algorithm 1. The alternative implementation with a priority queue has worse complexity in terms of the number of events in the full phylogeny but better complexity in terms of the size of the type space. In applications with large type spaces, the priority queue based implementation may be preferred. However, since our original algorithm has runtime that is strictly linear in the size of the full phylogeny, we focus on it for this work.

B Proof of Theorem 1

Consider a non-empty phylogeny $\mathcal{T} \neq T_\emptyset$. Let $p(\mathcal{T})$ be the probability density of observing $\mathcal{T}$ in the full model, $p(\mathcal{T} \mid \mathcal{T} \neq T_\emptyset)$ be the probability density of observing $\mathcal{T}$ in the full model conditional on the non-emptiness of $\mathcal{T}$, and let $p^{\mathsf{FE}}(\mathcal{T})$ be the probability density of observing $\mathcal{T}$ in the forward-equivalent model. We will show that $p(\mathcal{T} \mid \mathcal{T} \neq T_\emptyset)$ and $p^{\mathsf{FE}}(\mathcal{T})$ can both be computed by solving an ordinary differential equation. We will show $p(\mathcal{T} \mid \mathcal{T} \neq T_\emptyset) = p^{\mathsf{FE}}(\mathcal{T})$ by showing that these differential equations are the same. We draw on a large body of work which computes likelihoods in BDMS models using ordinary differential equations [15].

B.1 The non-observation probability

Eq. (2) agrees with (15) of [15] together with the modifications described in their appendix for handling concerted sampling events. Similar equations have appeared multiple time in earlier works; see [15] for references. For completeness, this section describes the derivation of these equations.

Between CSEs: For τ in an open interval between CSEs, consider a lineage of type a at time $\tau + \mathrm{d}\tau$. There are four ways that this lineage has no sampled descendants. We list this ways, and the probability of each, below.

1. No events occur in the interval $[\tau, \tau+\mathrm{d}\tau]$ and the lineage at time τ has no sampled descendants

$$\text{with probability } \Big(1 - \Big(\sum_{b=1}^{d} \lambda_{a,b}(\tau) + \mu_a(\tau) + \psi_a(\tau) + \sum_{b=1}^{d} \gamma_{a,b}(\tau)\Big)\mathrm{d}\tau\Big)E_a(\tau). \tag{8}$$

2. Birth $a \to (a, b)$ occurs in the interval $[\tau, \tau + \mathrm{d}\tau]$, and neither child has a sampled descendant

$$\text{with probability } \lambda_{a,b}(\tau)\mathrm{d}\tau E_a(\tau)E_b(\tau). \tag{9}$$

3. Death occurs in the interval $[\tau, \tau + \mathrm{d}\tau]$

$$\text{with probability } \mu_a(\tau)\mathrm{d}\tau. \tag{10}$$

4. Anagenetic mutation $a \to b$ occurs in the interval $[\tau, \tau + \mathrm{d}\tau]$, and the new lineage has not sampled descendants
$$\text{with probability } \gamma_{a,b}(\tau)\mathrm{d}\tau E_b(\tau). \tag{11}$$

Summing these possibilities (and noting that items 2 and 4 can occur for any $b = 1, \dots, d$) and rearranging gives the expression for $\frac{\mathrm{d}E_a(\tau)}{\mathrm{d}\tau}$ in (2).

Initialization: 0^- refers to the moment immediately after that CSE occurring at the present, after that sampling and death events for that CSE have occurred. There is no possibility for any further sampling, so $E_a(0^-) = 1$.

At CSEs: The equation $E_a(t_l^-) = \lim_{\tau\uparrow t_l} E_a(\tau)$ holds because the time t_l^- is after the sampling and death events for the CSE have occurred, so that $E_a(t_l^-)$ is the continuous extension of the extinction probability on the interval of time following the CSE. The time t_l^+ is immediately before the sampling and death events for the CSE have occurred. At this point in time, there is only one way in which the lineage can have no sampled descendants: the lineage is not sampled during the CSE (which has probability $1-\rho_{a,l}$), and the lineage following the CSE has no sampled descendants (which has probability $E_a(t_l^-)$). Thus, $E_a(t_l^+) = (1 - \rho_{a,l})E_a(t_l^-)$.

B.2 Unconditional probability density of the reconstructed phylogeny

Next, we describe how to compute $p(\mathcal{T})$ in the full model by solving a system of ordinary differential equations. This argument is provided in [15], which itself unifies arguments appearing many times previously. For completeness, we describe the argument here.

First, let us introduce some notation borrowed from [15]. The phylogeny $\mathcal{T}$ consists of a collection of edges $\mathcal{E}$, where an edge on the tree is defined as the segment spanning two adjacent events, where events are either birth events, anagenetic mutation events, sampling events at a non-CSEs, or CSEs (with the accompany sampling or death events). In the reconstructed phylogeny, death events are simply sampling events after which no further descendant lineages were sampled. Thus, sampling events that were followed by immediate death (whose probability is given by $\psi_a(\tau)$ and $r_a(\tau)$), and sampling events followed by survival but the failure to sample any future descendants, look the same. In the reconstructed phylogeny, sampling events can be internal to the tree or at leaf nodes, but all leaf nodes correspond to sampling events.

Each edge $e \in \mathcal{E}$ spans a time interval $s_e < \tau < t_e$. Letting a_e denote the type of the lineage along edge e, we let $g_e(\tau)$ denote the probability density that a lineage initialized at time τ with type a_e would give rise to the sub-tree of $\mathcal{T}$ "anchored" at time τ on edge e. We use this terminology to refer to the sub-tree whose root is at time τ on edge e and which contains all points on τ which are descendants of this point. The probability density $g_e(\tau)$ *does not* condition on non-emptiness.

The quantities $g_e(\tau)$ are given by the solution to an ODE which is integrated backwards in time along each edge, with the initial condition at s_e determined by the solution along the more recent adjacent edges.

ODE: The ODE is given by

$$\begin{aligned}\frac{\mathrm{d}g_e(\tau)}{\mathrm{d}\tau} = \lambda_{a,a}(\tau)\big(2E_a(\tau)-1\big)g_e(\tau) + \sum_{b\neq a}\lambda_{a,b}(\tau)\big(E_b(\tau)-1\big)g_e(\tau)\\ + \mu_a(\tau)\big(0-g_e(\tau)\big)\\ + \psi_a(\tau)\big(0-g_e(\tau)\big)\\ + \sum_{b\neq a}\gamma_{a,b}(\tau)(0-g_e(\tau)).\end{aligned} \tag{12}$$

To see this, consider the probability density of the sub-tree anchored at time $\tau + \mathrm{d}\tau$ on edge e. The probability of this tree is given by the product of the probability that a lineage of type a at time $\tau + \mathrm{d}\tau$ does not experience any observed events in the time interval $[\tau, \tau + \mathrm{d}\tau]$ and the probability density of the sub-tree anchored at time τ. There are three ways that no events are observed in the time interval $[\tau, \tau + \mathrm{d}\tau]$. The first way is that no events occur. This has probability $1 - \Big(\sum_{b=1}^{d}\lambda_{a,b}(\tau) + \mu_a(\tau) + \psi_a(\tau) + \sum_{b\neq a}\gamma_{a,b}(\tau)\Big)\mathrm{d}\tau$. The second is that a birth of type $a \to a, a$ occurs and one of the child lineages has no sampled descendants. This has probability $2\lambda_{a,a}(\tau)\mathrm{d}\tau E_a(\tau)$, where the factor of 2 results from the possibility that either lineage is the one without sampled descendants. The third way is that a birth $a \to (a, b)$ for $b \neq a$ occurs and the child lineage of type b has no sampled descendants. This has probability $\lambda_{a,b}(\tau)\mathrm{d}\tau E_b(\tau)$. Thus,

$$g_e(\tau+\mathrm{d}\tau) = \Big(1-\Big(\sum_{b=1}^{d}\lambda_{a,b}(\tau)+\mu_a(\tau)+\psi_a(\tau)+\sum_{b\neq a}\gamma_{a,b}(\tau)\Big)\mathrm{d}\tau+2\lambda_{a,a}(\tau)\mathrm{d}\tau E_a(\tau)+\sum_{b\neq a}\lambda_{a,b}(\tau)\mathrm{d}\tau E_b(\tau)\Big)g_e(\tau). \tag{13}$$

This rearranges to (12).

Initial conditions: The initial condition at s_e depends on the type of event occurring at the terminal node of an edge.

- **Births:** For birth events resulting in edges (e_1, e_2) with types (a, b) or (b, a),

$$g_e(s_e) = \lambda_{a,b}(s_e)g_{e_1}(s_e)g_{e_2}(s_e). \tag{14a}$$

  The factor $\lambda_{a,b}(s_e)$ gives the probability density of a birth occurring at time τ, and $g_{e_1}(s_e)g_{e_2}(s_e)$ the probability of the two children giving rise to their observed sub-trees.

- **Anagenetic mutations:** For anagenetic mutations $a \to b$ with offspring edge e_1,

$$g_e(s_e) = \big(\gamma_{a,b}(s_e) + \lambda_{a,b}(s_e)E_a(s_e)\big)g_{e_1}(s_e). \tag{14b}$$

  An anagenetic mutation $a \to b$ in the reconstructed phylogeny can result from two types of events in the full phylogeny. The first type of event is the anagenetic mutation $a \to b$ in the full phylogeny with the child lineage giving rise to the observed sub-tree. This has probability density $\gamma_{a,b}(s_e)g_{e_1}(s_e)$. The second type of event is a birth $a \to (a, b)$, with the child of type a having no observed descendants and the child of type b giving rise to the observed sub-tree. This has probability density $\lambda_{a,b}(s_e)E_a(s_e)g_{e_1}(s_e)$. Adding these two terms gives (14b).

- **Non-CSE sampling events:** These are initialized by

$$g_e(s_e) = \begin{cases} \psi_a(s_e)(r_a(s_e) + (1 - r_a(s_e))E_a(s_e)) & \text{for leaf nodes,} \\ \psi_a(s_e)(1 - r_a(s_e))g_{e_1}(s_e) & \text{for internal nodes.} \end{cases} \tag{14c}$$

  A sampled leaf node at a non-CSE time can result from two types of events in the full phylogeny. The first type is that the lineage was sampled and immediately went extinct. This has probability density $\psi_a(s_e)r_a(s_e)$. The second type is that the lineage was sampled, did not immediately go extinct, and then had no sampled descendants. This has probability density $\psi_a(s_e)(1 - r_a(s_e))E_a(s_e)$. Adding these two terms gives the first line of (14c).

  A sampled internal node at a non-CSE time can result from only one type of event in the full phylogeny. This event is that the lineage was sampled, did not immediately go extinct, and then gave rise to the observed sub-tree. This has probability density $\psi_a(s_e)(1-r_a(s_e))g_{e_1}(s_e)$, giving the second line of (14c).

- **CSE sampling events:** These are initialized by

$$g_e(t_l^+) = \begin{cases} (1 - \rho_{a,l})g_{e_1}(t_l^-) & \text{for non-sampled nodes,} \\ \rho_{a,l}(r_{a,l} + (1 - r_{a,l})E_a(t_l^-)) & \text{for sampled leaf nodes,} \\ \rho_{a,l}(1 - r_{a,l})g_{e_1}(t_l^-) & \text{for sampled internal nodes.} \end{cases} \tag{14d}$$

  If no sampling occurs at the CSE in the reconstructed phylogeny, then no sampling occurred in the full phylogeny and the lineage at time t_l^- gave rise to the observed sub-tree. This has probability density $(1 - \rho_{a,l})g_{e_1}(t_l^-)$, giving the first line of (14d). The remaining two lines of (14d) follow by exactly the same argument leading to the two lines of (14c), with $\psi_a(s_e)$ replaced by $\rho_{a,l}$ and $r_a(s_e)$ replaced by $r_{a,l}$.

One initializes these ODEs at every leaf node in the reconstructed phylogeny, and then integrates backwards in time along all lineages until reaching the root. The value $g_e(t_{\max})$ on the root edge of the tree is the probability density of observing $\mathcal{T}$.

B.3 Probability density conditional on non-emptiness

For an edge e and time τ along edge e in $\mathcal{T}$, let $\overline{g}_e(\tau)$ denote the probability density that a lineage initialized at time τ with type a_e gives rise to the sub-tree of $\mathcal{T}$ anchored at time τ on edge e, conditional on such a lineage having an observed descendant. That is, according to Bayes rule,

$$\overline{g}_e(\tau) = \frac{g_e(\tau)}{1 - E_a(\tau)}. \tag{15}$$

Let $g_e^{\mathsf{FE}}(\tau)$ denote the quantity $g_e(\tau)$ for the forward equivalent model. That is, $g_e^{\mathsf{FE}}(\tau)$ is defined using parameters $(\pi^{\mathsf{FE}}, \lambda_{a,b}^{\mathsf{FE}}, \mu_a^{\mathsf{FE}}, \psi_a^{\mathsf{FE}}, r_a^{\mathsf{FE}}, \gamma_{a,b}^{\mathsf{FE}}, \rho_{a,l}^{\mathsf{FE}}, r_{a,l}^{\mathsf{FE}})$ in (12) and (14), with $E_a^{\mathsf{FE}}(\tau)$ as determined by these parameters according to Section B.1. Denoting by e_* the root lineage of $\mathcal{T}$, Theorem 1 will follow by establishing

$$\overline{g}_{e_*}(t_{\max}) = g_{e_*}^{\mathsf{FE}}(t_{\max}). \tag{16}$$

The remainder of the proof consists of establishing (16). This is done by showing that $\overline{g}_e(\tau)$ obeys the same ODEs and initial conditions as $g_e^{\mathsf{FE}}(\tau)$, given by (12) and (14) with the forward-equivalent model parameters.

First, we collect some facts about the forward-equivalent model parameters. By (3), $r^{\mathsf{FE}}_{a,0} = r_{a,0} + (1 - r_{a,0})E_a(0^-) = 1$ and $\rho^{\mathsf{FE}}_{a,0} = \rho_{a,0}/(1 - E_a(0^+)) = 1$. As explained in Section B.1, $E^{\mathsf{FE}}_a(0^-) = 1$. Thus, $E^{\mathsf{FE}}_a(0^+) = (1-\rho^{\mathsf{FE}}_{a,0})E^{\mathsf{FE}}_a(0^-) = 0$. Then, because $\mu^{\mathsf{FE}}_a(0) = 0$, Eqs. (2) and (2) imply $E^{\mathsf{FE}}_a(\tau) = 0$ for all $\tau \geq 0$ (excluding $E^{\mathsf{FE}}_a(0^-)$). We collect these identities in the next display:

$$\rho^{\mathsf{FE}}_{a,0} = 1, \quad \rho^{\mathsf{FE}}_{a,0} = 1, \quad E^{\mathsf{FE}}_a(0^-) = 1, \quad E^{\mathsf{FE}}_a(\tau) = 0 \text{ for } \tau > 0^-. \tag{17}$$

Agreement of ODEs. We differentiate (15) to get the ODE satisfied by $\overline{g}_e(\tau)$. In particular,

$$\frac{\mathrm{d}\overline{g}_e(\tau)}{\mathrm{d}\tau} = \frac{\frac{\mathrm{d}}{\mathrm{d}\tau}g_e(\tau)}{1 - E_a(\tau)} + \frac{g_e(\tau)}{(1 - E_a(\tau))^2}\frac{\mathrm{d}E_a(\tau)}{\mathrm{d}\tau}. \tag{18}$$

Using (2) and (12), this is

$$\begin{aligned}
=&\frac{(\lambda_{a,a}(\tau)E_a(\tau) + \sum_b \lambda_{a,b}(\tau)(E_b(\tau) - 1) - \mu_a(\tau) - \psi_a(\tau) - \sum_b \gamma_{a,b}(\tau))g_e(\tau)}{1 - E_a(\tau)} \\
&+ \frac{g_e(\tau)}{(1 - E_a(\tau))^2}\Big(\sum_b \lambda_{a,b}(\tau)(E_a(\tau)E_b(\tau) - E_a(\tau)) + \mu_a(\tau)(1 - E_a(\tau)) \\
&\qquad + \psi_a(\tau)(0 - E_a(\tau)) + \sum_b \gamma_{a,b}(\tau)(E_b(\tau) - E_a(\tau))\Big).
\end{aligned} \tag{19}$$

The terms involving $\lambda_{a,b}(\tau)$ are $g_e(\tau)/(1 - E_a(\tau))$ multiplied by

$$\begin{aligned}
&\lambda_{a,a}(\tau)E_a(\tau) + \sum_b \lambda_{a,b}(\tau)(E_b(\tau) - 1) + \frac{\sum_b \lambda_{a,b}(\tau)(E_a(\tau)E_b(\tau) - E_a(\tau))}{1 - E_a(\tau)} \\
&= \lambda_{a,a}(\tau)E_a(\tau) - \sum_b \lambda^{\mathsf{FE}}_{a,b}(\tau) - \sum_b \frac{1 - E_b(\tau)}{1 - E_a(\tau)}E_a(\tau)\lambda_{a,b}(\tau).
\end{aligned} \tag{20}$$

The terms involving $\mu_a(\tau)$ are $g_e(\tau)/(1 - E_a(\tau))$ multiplied by

$$-\mu_a(\tau) + \frac{\mu_a(\tau)(1 - E_a(\tau))}{1 - E_a(\tau)} = 0 = \mu^{\mathsf{FE}}_a(\tau). \tag{21}$$

The terms involving $\psi_a(\tau)$ are $g_e(\tau)/(1 - E_a(\tau))$ multiplied by

$$-\psi_a(\tau) - \psi_a(\tau)\frac{E_a(\tau)}{1 - E_a(\tau)} = -\psi^{\mathsf{FE}}_a(\tau). \tag{22}$$

The terms involving $\gamma_{a,b}(\tau)$ are $g_e(\tau)/(1 - E_a(\tau))$ multiplied by

$$-\sum_b \gamma_{a,b}(\tau) + \frac{\sum_b \gamma_{a,b}(\tau)(E_b(\tau) - E_a(\tau))}{1 - E_a(\tau)} = -\sum_b \frac{1 - E_b(\tau)}{1 - E_a(\tau)}\gamma_{a,b}(\tau). \tag{23}$$

Combining these calculations,

$$\begin{aligned}
&\frac{\mathrm{d}\overline{g}_e(\tau)}{\mathrm{d}\tau} \\
&= \Big(-\sum_b \lambda^{\mathsf{FE}}_{a,b}(\tau) - \mu^{\mathsf{FE}}_a(\tau) - \psi^{\mathsf{FE}}_a(\tau) - \sum_b \frac{1 - E_b(\tau)}{1 - E_a(\tau)}(\gamma_{a,b}(\tau) + E_a(\tau)\lambda_{a,b}(\tau)) + \lambda_{a,a}(\tau)E_a(\tau)\Big)\overline{g}_e(\tau) \\
&= \Big(-\sum_b \lambda^{\mathsf{FE}}_{a,b}(\tau) - \mu^{\mathsf{FE}}_a(\tau) - \psi^{\mathsf{FE}}_a(\tau) - \sum_{b\neq a} \frac{1 - E_b(\tau)}{1 - E_a(\tau)}(\gamma_{a,b}(\tau) + E_a(\tau)\lambda_{a,b}(\tau))\Big)\overline{g}_e(\tau) \\
&= \Big(-\sum_b \lambda^{\mathsf{FE}}_{a,b}(\tau) - \mu^{\mathsf{FE}}_a(\tau) - \psi^{\mathsf{FE}}_a(\tau) - \sum_{b\neq a} \gamma^{\mathsf{FE}}_{a,b}(\tau)\Big)\overline{g}_e(\tau).
\end{aligned} \tag{24}$$

where the second inequality uses that $(1 - E_a(\tau))/(1 - E_a(\tau)) = 1$ and $\gamma_{a,a}(\tau) = 0$. Because $E_a^{\mathsf{FE}}(\tau) = 0$ for all $\tau \geq 0$, this ODE is (12) under the forward-equivalent model parameters.

Agreement of initial conditions. We now check agreement of the initial conditions.

- **Births:** For birth events resulting in edges (e_1, e_2) with types (a, b) or (b, a), Bayes rule and (14a) give

$$\begin{aligned}\overline{g}_e(s_e) &= \frac{g_e(s_e)}{1 - E_a(s_e)} = \frac{\lambda_{a,b}(\tau) g_{e_1}(s_e) g_{e_2}(s_e)}{1 - E_a(s_e)} = \lambda_{a,b}(\tau)(1 - E_b(s_e)) \frac{g_{e_1}(s_e)}{1 - E_a(s_e)} \frac{g_{e_2}(s_e)}{1 - E_b(s_e)} \\ &= \lambda_{a,b}^{\mathsf{FE}}(\tau) \overline{g}_{e_1}(\tau) \overline{g}_{e_2}(\tau),\end{aligned} \tag{25}$$

  which is (14a) for the forward-equivalent model.

- **Anagenetic mutations:** For anagenetic mutations $a \to b$ with offspring edge e_1, Bayes rule and (14b) give

$$\begin{aligned}\overline{g}_e(s_e) &= \frac{g_e(s_e)}{1 - E_a(s_e)} = \frac{\gamma_{a,b}(s_e) + \lambda_{a,b}(s_e) E_a(s_e)}{1 - E_a(s_e)} g_{e_1}(s_e) \\ &= \frac{(1 - E_b(s_e))(\gamma_{a,b}(s_e) + \lambda_{a,b}(s_e) E_a(s_e))}{1 - E_a(s_e)} \frac{g_{e_1}(s_e)}{1 - E_b(s_e)} = \gamma_{a,b}^{\mathsf{FE}}(s_e) \overline{g}_{e_1}(s_e),\end{aligned} \tag{26}$$

  which is (14b) for the forward-equivalent model.

- **Non-CSE sampling events:** At leaf nodes,

$$\begin{aligned}\overline{g}_e(s_e) &= \frac{g_e(s_e)}{1 - E_a(s_e)} = \frac{\psi_a(s_e)(r_a(s_e) + (1 - r_a(s_e)) E_a(s_e))}{1 - E_a(s_e)} \\ &= \psi_a^{\mathsf{FE}}(s_e)(r_a^{\mathsf{FE}}(s_e) + (1 - r_a^{\mathsf{FE}}(s_e)) E_a^{\mathsf{FE}}(s_e)),\end{aligned} \tag{27}$$

  which is the first line of (14c) for the forward-equivalent model. At internal nodes,

$$\begin{aligned}\overline{g}_e(s_e) &= \frac{g_e(s_e)}{1 - E_a(s_e)} = \frac{\psi_a(s_e)(1 - r_a(s_e)) g_{e_1}(s_e)}{1 - E_a(s_e)} \\ &= \frac{\psi_a(s_e)}{1 - E_a(s_e)} (1 - E_a(s_e))(1 - r_a(s_e)) \frac{g_{e_1}(s_e)}{1 - E_a(s_e)} \\ &= \psi_a^{\mathsf{FE}}(s_e)(1 - r_a^{\mathsf{FE}}(s_e)) g_{e_1}^{\mathsf{FE}}(s_e),\end{aligned} \tag{28}$$

  which is the second line of (14c) for the forward equivalent model.

- **CSE sampling events:** Using $\rho_{a,l}^{\mathsf{FE}} = \rho_{a,l}/(1 - E_a(t_l^+))$ (by (3)) and $E_a(t_l^+) = (1 - r_{a,l}) E_a(t_l^-)$ (by (2)), that $1 - \rho_{a,l} = \frac{1-\rho_{a,l}^{\mathsf{FE}}}{1-\rho_{a,l}^{\mathsf{FE}} E(t_l^-)}$. Then, for non-sampling at CSE times,

$$\begin{aligned}\overline{g}_e(t_l^+) &= \frac{g_e(t_l^+)}{1 - E_a(t_l^+)} = \frac{(1 - \rho_{a,l}) g_{e_1}(t_l^-)}{1 - (1 - \rho_{a,l}) E_1(t_l^-)} \\ &= \frac{\frac{1-\rho_{a,l}^{\mathsf{FE}}}{1-\rho_{a,l}^{\mathsf{FE}} E(t_l^-)} g_{e_1}(t_l^-)}{1 - \frac{1-\rho_{a,l}^{\mathsf{FE}}}{1-\rho_{a,l}^{\mathsf{FE}} E(t_l^-)} E_1(t_l^-)} = (1 - \rho_{a,l}^{\mathsf{FE}}) \frac{g_{e_1}(t_l^-)}{1 - E_1(t_l^-)} = (1 - \rho_{a,l}^{\mathsf{FE}}) \overline{g}_{e_1}(t_l^-),\end{aligned} \tag{29}$$

which is the first line of (14d) for the forward equivalent model. For sampled leaf nodes at CSE times,

$$\overline{g}_e(t_l^+) = \frac{g_e(t_l^+)}{1 - E_a(t_l^+)} = \frac{\rho_{a,l}(r_{a,l} + (1 - r_{a,l})E_a(t_l^-))}{1 - E_a(t_l^+)} = \rho_{a,l}^{\mathsf{FE}} r_{a,l}^{\mathsf{FE}}, \tag{30}$$

which is the second line of (14d) for the forward equivalent model (recall $E_a^{\mathsf{FE}}(t_l^+) = 0$). For sampled internal nodes at CSE times,

$$\overline{g}_e(t_l^+) = \frac{g_e(t_l^+)}{1 - E_a(t_l^+)} = \frac{\rho_{a,l}(1 - r_{a,l})g_{e_1}(t_l^-)}{1 - E_a(t_l^+)} = \rho_{a,l}^{\mathsf{FE}}(1 - r_{a,l}^{\mathsf{FE}})\frac{g_{e_1}(t_l^-)}{1 - E_a(t_l^-)} = \rho_{a,l}^{\mathsf{FE}}(1 - r_{a,l}^{\mathsf{FE}})\overline{g}_{e_1}(t_l^-). \tag{31}$$

where we have used $1 - r_{a,l}^{\mathsf{FE}} = (1 - r_{a,l})(1 - E_a(t_l^-))$ by (3). This is the third line of (14d) for the forward equivalent model.

Thus, we have confirmed that $\overline{g}_e(\tau)$ and $g_e^{\mathsf{FE}}(\tau)$ obey the same ODE and initial conditions. We conclude (16), which completes the proof of Theorem 1.

C Proof of Proposition 2 and Proposition 1

For a tree T, let #nodes(T) be the number of nodes of T. In the context of the BDMS model, nodes correspond to (1) birth events, (2) death (or extinction) events, (3) mutation events, and (4) sampling events. With suitable preprocessing, the runtime required to simulate a full tree T from a given BDMS model is (up to a constant) exactly #nodes(T). We mean 'full' tree T, because the 'observed' or 'reconstructed' tree, denoted $r(T)$, is instead the sub-tree induced by the sampled leaves, and usually the desired output from the simulation. Since a full tree might have an empty reconstructed tree, the time required to produce a non-empty reconstructed tree from the original BDMS model via retrying is #nodes(T_1) + #nodes(T_2) + $\cdots$ + #nodes(T_K) where $T_1, T_2, \ldots$ is an infinite sequence of full trees simulated i.i.d. from the BDMS model and T_K is the first full tree with non-empty reconstruction (so that K is a stopping time random variable). As we have shown, given a BDMS model, there exists an equivalent BDMS model—in the sense of providing the same distribution over reconstructed trees conditional on non-emptyness—such that under the new BDMS model there is no death ($\mu_a^{\mathsf{FE}}(\tau) = 0$) and all leaves are sampled. In other words, the new model can simulate exactly from the original BDMS model *without having to perform any retries nor reconstruction.* The runtime of the forward-equivalent BDMS model (under the obvious coupling) is hence just #nodes($r(T_K)$) rather than #nodes(T_1) + #nodes(T_2) + $\cdots$ + #nodes(T_K), which may be orders of magnitudes faster if $r(T_K)$ is significantly smaller than T_K or if a lot of retries are needed (i.e., if K is large). Let $\mathbb{P}, \mathbb{E}$ be probabilities and expectations under the original BDMS model, and let $\mathbb{P}^{\mathsf{FE}}, \mathbb{E}^{\mathsf{FE}}$ be probabilities and expectations under the forward-equivalent BDMS model. The expected runtime of the original BDMS model is:

$$R = \mathbb{E}[\#\text{nodes}(T_1) + \#\text{nodes}(T_2) + \cdots + \#\text{nodes}(T_K)]$$

whereas the expected runtime of the forward-equivalent BDMS model is:

$$R^{\mathsf{FE}} = \mathbb{E}[\#\text{nodes}(r(T_K))]$$

It is immediately clear that $R \geq R^{\mathsf{FE}}$, i.e., it is faster to simulate under the forward-equivalent model. Our Proposition 2 gives a more precise characterization of this speedup, in a way that is easy to compute with time discretization and dynamic programming. We now prove the Lemma.

Proof of Proposition 2. Under our setup, the runtime of the forward-equivalent model is:

$$R^{\mathsf{FE}} = \mathbb{E}[\#\text{nodes}(r(T_K))] = \mathbb{E}[\#\text{nodes}(r(T_1))|r(T_1) \neq T_\emptyset]$$

As for the runtime of the original BDMS model, let $S = \#\text{nodes}(T_1) + \#\text{nodes}(T_2) + \cdots + \#\text{nodes}(T_K)$. By total expectation, we have that:

$$\begin{aligned}R =&\mathbb{E}[S]\\ =&\mathbb{P}(r(T_1) = T_\emptyset)\mathbb{E}[S|r(T_1) = T_\emptyset]\\ &+ \mathbb{P}(r(T_1) \neq T_\emptyset)\mathbb{E}[S|r(T_1) \neq T_\emptyset] =\\ =&\mathbb{P}(r(T_1) = T_\emptyset)(\mathbb{E}[\#\text{nodes}(T_1)|r(T_1) = T_\emptyset] + R)\\ &+ \mathbb{P}(r(T_1) \neq T_\emptyset)\mathbb{E}[\#\text{nodes}(T_1)|r(T_1) \neq T_\emptyset]\\ =&\mathbb{P}(r(T_1) = T_\emptyset)R + \mathbb{E}[\#\text{nodes}(T_1)]\end{aligned}$$

so that solving for R we get:

$$R = \frac{\mathbb{E}[\#\text{nodes}(T_1)]}{\mathbb{P}(r(T_1) \neq T_\emptyset)}$$

Another way to show this is by noting that the runtime of tree T_i should be considered if and only if all of $r(T_1), \ldots, r(T_{i-1})$ are empty. Crucially, for fixed i, whether we consider the runtime from tree T_i does not depend on whether T_i is empty or not. (Note that this is not true for T_K; the runtime of T_K is biased by the fact that it is, by definition, non-empty.) Hence,

$$R = \sum_{i=1}^{\infty} \mathbb{P}(r(T_1) = T_\emptyset)^{i-1}\mathbb{E}[\#\text{nodes}(T_i)] = \frac{\mathbb{E}[\#\text{nodes}(T_1)]}{\mathbb{P}(r(T_1) \neq T_\emptyset)}$$

Combining our identities for R and R^{FE} we obtain:

$$\frac{R}{R^{\mathsf{FE}}} = \frac{\mathbb{E}[\#\text{nodes}(T_1)]}{\mathbb{P}(r(T_1) \neq T_\emptyset)\mathbb{E}[\#\text{nodes}(r(T_1))|r(T_1) \neq T_\emptyset]}$$

Since $\mathbb{E}[\#\text{nodes}(r(T_1))|r(T_1) = T_\emptyset] = 0$, we can complete the denominator using the law of total expectation to obtain:

$$\frac{R}{R^{\mathsf{FE}}} = \frac{\mathbb{E}[\#\text{nodes}(T_1)]}{\mathbb{E}[\#\text{nodes}(r(T_1))]}$$

as claimed. □

With this, it is easy to prove Proposition 1:

Proof of Proposition 1. We compute $\mathbb{E}[\#\text{nodes}(T)]$ and $\mathbb{E}[\#\text{nodes}(r(T))]$ and take the ratio, which by the lemma provides the speedup. Note that

$$\mathbb{E}[\#\text{nodes}(r(T))] = 2\rho\mathbb{E}[\text{number of leaf nodes}]$$

and

$$\mathbb{E}[\#\text{nodes}(T)] = 2\mathbb{E}[\text{number of leaf nodes}] + 2\mathbb{E}[\text{number of deaths}]$$

so it suffices to compute $\mathbb{E}$[number of leaf nodes] and $\mathbb{E}$[number of deaths], which we do separately. Each of these quantities is given by an ODE; let:

$$l(t) = \mathbb{E}[\text{number of leaf nodes}|\text{start with one individual at time } t]$$

and

$$d(t) = \mathbb{E}[\text{number of deaths}|\text{start with one individual at time } t]$$

then:

$$\dot{l}(t) = (\lambda - \mu)l(t); \quad l(0) = 1$$
$$\dot{d}(t) = \mu + (\lambda - \mu)d(t); \quad d(0) = 0$$

If $\lambda = \mu$ then the solutions are $l(t) = 1, d(t) = t\mu$. Otherwise:

$$l(t) = e^{(\lambda-\mu)t}$$
$$d(t) = \frac{\mu(e^{(\lambda-\mu)t} - 1)}{\lambda - \mu}$$

Evaluating at $t = 1$ provides the desired quantities from which we can derive the given ratio.

References

[1] P. Barrat-Charlaix, J. Huddleston, T. Bedford, and R. A. Neher. Limited Predictability of Amino Acid Substitutions in Seasonal Influenza Viruses. *Molecular Biology and Evolution*, 38(7):2767–2777, 03 2021.

[2] G. E. Budd and R. P. Mann. History is written by the victors: The effect of the push of the past on the fossil record. *Evolution*, 72(11):2276–2291, Sept. 2018.

[3] W. S. DeWitt, M. Celentano, T. Bakis, D. K. Ralph, S. Prillo, F. A. Matsen, IV, Y. S. Song, and V. M. Minin. BDMS: A tree simulator for birth-death-mutation-sampling processes, 2024. https://doi.org/10.5281/zenodo.10467425.

[4] R. G. FitzJohn, W. P. Maddison, and S. P. Otto. Estimating Trait-Dependent Speciation and Extinction Rates from Incompletely Resolved Phylogenies. *Systematic Biology*, 58(6):595–611, 10 2009.

[5] K. B. Hoehn and S. H. Kleinstein. B cell phylogenetics in the single cell era. *Trends Immunol.*, 0(0), Dec. 2023.

[6] S. Höhna, T. Stadler, F. Ronquist, and T. Britton. Inferring Speciation and Extinction Rates under Different Sampling Schemes. *Molecular Biology and Evolution*, 28(9):2577–2589, 04 2011.

[7] F. Horns, C. Vollmers, D. Croote, S. F. Mackey, G. E. Swan, C. L. Dekker, M. M. Davis, and S. R. Quake. Lineage tracing of human B cells reveals the in vivo landscape of human antibody class switching. *eLife*, 5:e16578, aug 2016.

[8] M. B. Horton, H. Cheon, K. R. Duffy, D. Brown, S. H. Naik, C. Alvarado, J. R. Groom, S. Heinzel, and P. D. Hodgkin. Lineage tracing reveals b cell antibody class switching is stochastic, cell-autonomous, and tuneable. *Immunity*, 55(10):1843–1855.e6, Oct 2022.

[9] J. Huerta-Cepas, F. Serra, and P. Bork. ETE 3: Reconstruction, Analysis, and Visualization of Phylogenomic Data. *Molecular Biology and Evolution*, 33(6):1635–1638, 02 2016.

[10] M. Jones, A. Khodaverdian, J. Quinn, M. Chan, J. Hussmann, R. Wang, C. Xu, J. Weissman, and N. Yosef. Inference of single-cell phylogenies from lineage tracing data using cassiopeia. *Genome Biology*, 21(1), Apr. 2020. Publisher Copyright: © 2020 The Author(s).

[11] G. Laudanno, B. Haegeman, D. L. Rabosky, and R. S. Etienne. Detecting lineage-specific shifts in diversification: A proper likelihood approach. *Systematic Biology*, 70(2):389–407, 07 2020.

[12] B. Legried and J. Terhorst. A class of identifiable phylogenetic birth–death models. *Proceedings of the National Academy of Sciences*, 119(35):e2119513119, Aug. 2022.

[13] B. Legried and J. Terhorst. Identifiability and inference of phylogenetic birth–death models. *Journal of Theoretical Biology*, 568:111520, July 2023.

[14] S. Louca and M. W. Pennell. Extant timetrees are consistent with a myriad of diversification histories. *Nature*, 580(7804):502–505, 2020.

[15] A. MacPherson, S. Louca, A. McLaughlin, J. B. Joy, and M. W. Pennell. Unifying Phylogenetic Birth–Death Models in Epidemiology and Macroevolution. *Systematic Biology*, 71(1):172–189, 06 2021.

[16] W. P. Maddison, P. E. Midford, and S. P. Otto. Estimating a Binary Character's Effect on Speciation and Extinction. *Systematic Biology*, 56(5):701–710, 10 2007.

[17] B. R. Moore, S. Höhna, M. R. May, B. Rannala, and J. P. Huelsenbeck. Critically evaluating the theory and performance of bayesian analysis of macroevolutionary mixtures. *Proceedings of the National Academy of Sciences*, 113(34):9569–9574, 2016.

[18] H. Morlon. Phylogenetic approaches for studying diversification. *Ecology Letters*, 17(4):508–525, 2014.

[19] H. Morlon, T. L. Parsons, and J. B. Plotkin. Reconciling molecular phylogenies with the fossil record. *Proceedings of the National Academy of Sciences*, 108(39):16327–16332, 2011.

[20] S. Nee, R. M. May, and P. H. Harvey. The reconstructed evolutionary process. *Philosophical Transactions: Biological Sciences*, 344(1309):305–311, 1994.

[21] R. A. Neher and T. Bedford. nextflu: real-time tracking of seasonal influenza virus evolution in humans. *Bioinformatics*, 31(21):3546–3548, 06 2015.

[22] R. A. Neher and O. Hallatschek. Genealogies of rapidly adapting populations. *Proceedings of the National Academy of Sciences*, 110(2):437–442, 2013.

[23] R. A. Neher, C. A. Russell, and B. I. Shraiman. Predicting evolution from the shape of genealogical trees. *eLife*, 3:e03568, nov 2014.

[24] S. Prillo, A. Ravoor, N. Yosef, and Y. S. Song. ConvexML: Scalable and accurate inference of single-cell chronograms from CRISPR/Cas9 lineage tracing data, Dec. 2023.

[25] J. Quinn, M. Jones, R. Okimoto, S. Nanjo, M. Chan, N. Yosef, T. Bivona, and J. Weissman. Single-cell lineages reveal the rates, routes, and drivers of metastasis in cancer xenografts. *Science*, 371:eabc1944, 01 2021.

[26] J. J. Quinn, M. G. Jones, R. A. Okimoto, S. Nanjo, M. M. Chan, N. Yosef, T. G. Bivona, and J. S. Weissman. Single-cell lineages reveal the rates, routes, and drivers of metastasis in cancer xenografts. *Science*, 371(6532):eabc1944, 2021.

[27] M. A. Rolfes, I. M. Foppa, S. Garg, B. Flannery, L. Brammer, J. A. Singleton, E. Burns, D. Jernigan, S. J. Olsen, J. Bresee, and C. Reed. Annual estimates of the burden of seasonal influenza in the united states: A tool for strengthening influenza surveillance and preparedness. *Influenza Other Respir Viruses*, 12(1):132–137, Feb. 2018.

[28] K. P. Simeonov, C. N. Byrns, M. L. Clark, R. J. Norgard, B. Martin, B. Z. Stanger, J. Shendure, A. McKenna, and C. J. Lengner. Single-cell lineage tracing of metastatic cancer reveals selection of hybrid emt states. *Cancer Cell*, 39(8):1150–1162.e9, 2021.

[29] T. Stadler. Sampling-through-time in birth–death trees. *Journal of Theoretical Biology*, 267(3):396–404, 2010.

[30] T. Stadler, R. Kouyos, V. von Wyl, S. Yerly, J. Böni, P. Bürgisser, T. Klimkait, B. Joos, P. Rieder, D. Xie, H. F. Günthard, A. J. Drummond, S. Bonhoeffer, and the Swiss HIV Cohort Study. Estimating the Basic Reproductive Number from Viral Sequence Data. *Molecular Biology and Evolution*, 29(1):347–357, 09 2011.

[31] A. Thompson, B. Liebeskind, E. J. Scully, and M. Landis. Deep learning and likelihood approaches for viral phylogeography converge on the same answers whether the inference model is right or wrong. *bioRxiv*, 2023.

[32] P. O. Title and D. L. Rabosky. Tip rates, phylogenies and diversification: What are we estimating, and how good are the estimates? *Methods in Ecology and Evolution*, 10(6):821–834, 2019.

[33] S. Xie, L. Valente, and R. S. Etienne. Inferring state-dependent diversification rates using approximate bayesian computation (abc). *bioRxiv*, 2023.

[34] D. Yang, M. G. Jones, S. Naranjo, W. M. Rideout, K. H. J. Min, R. Ho, W. Wu, J. M. Replogle, J. L. Page, J. J. Quinn, F. Horns, X. Qiu, M. Z. Chen, W. A. Freed-Pastor, C. S. McGinnis, D. M. Patterson, Z. J. Gartner, E. D. Chow, T. G. Bivona, M. M. Chan, N. Yosef, T. Jacks, and J. S. Weissman. Lineage tracing reveals the phylodynamics, plasticity, and paths of tumor evolution. *Cell*, 185(11):1905–1923.e25, 2022.

[35] D. Yang, M. G. Jones, S. Naranjo, W. M. Rideout III, K. H. J. Min, R. Ho, W. Wu, J. M. Replogle, J. L. Page, J. J. Quinn, F. Horns, X. Qiu, M. Z. Chen, W. A. Freed-Pastor, C. S. McGinnis, D. M. Patterson, Z. J. Gartner, E. D. Chow, T. G. Bivona, M. M. Chan, N. Yosef, T. Jacks, and J. S. Weissman. Lineage tracing reveals the phylodynamics, plasticity, and paths of tumor evolution. *Cell*, 185(11):1905–1923.e25, May 2022.

Supplementary Figures

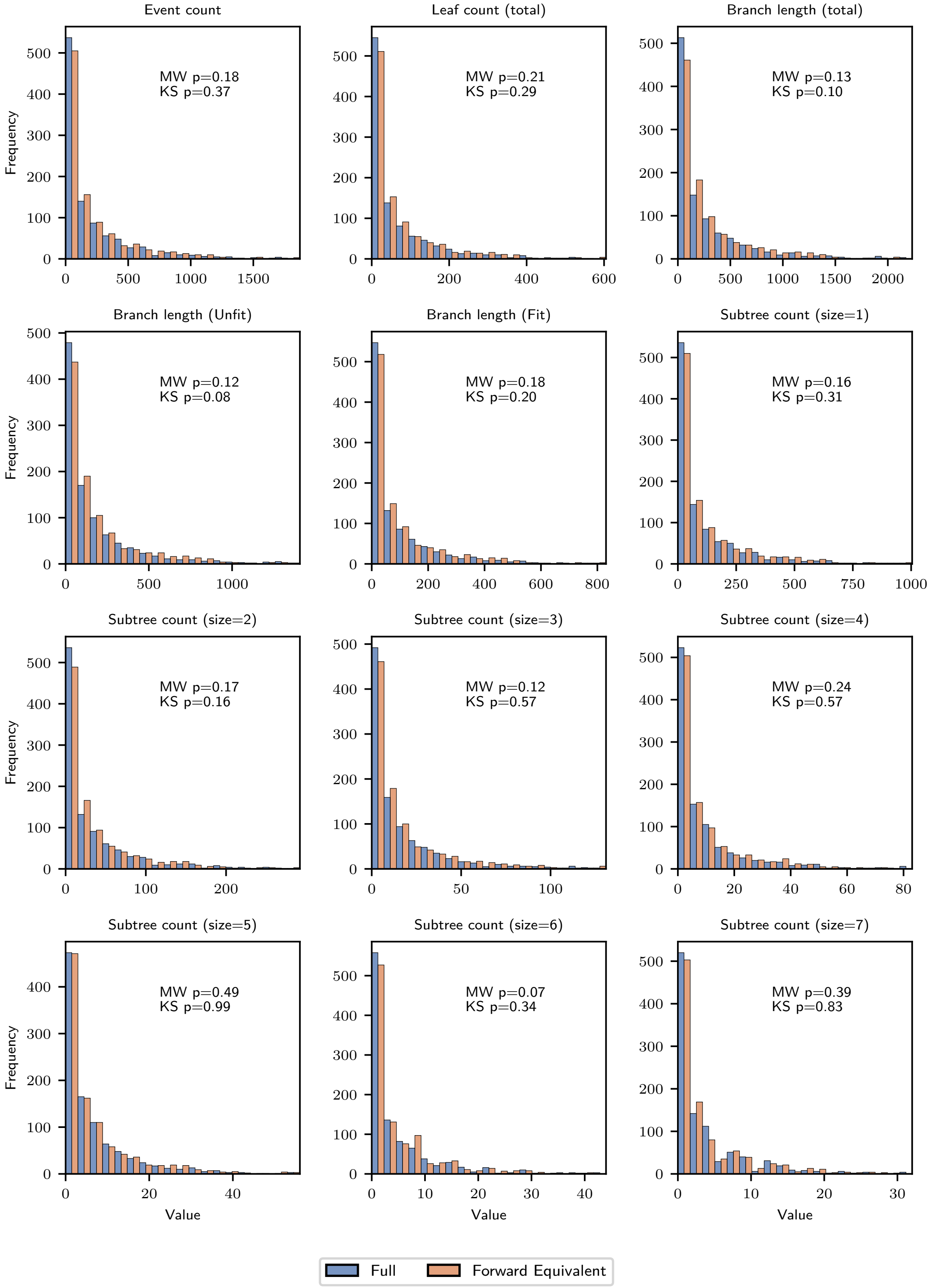

Figure 6: Distributional comparisons analogous to those appearing in Figure 3, for all summary statistics we considered, as described in Section 4.1. We emphasize that because all of these tests were computed using the same sample of phylogenies, the p-values for different tests need not be independent.

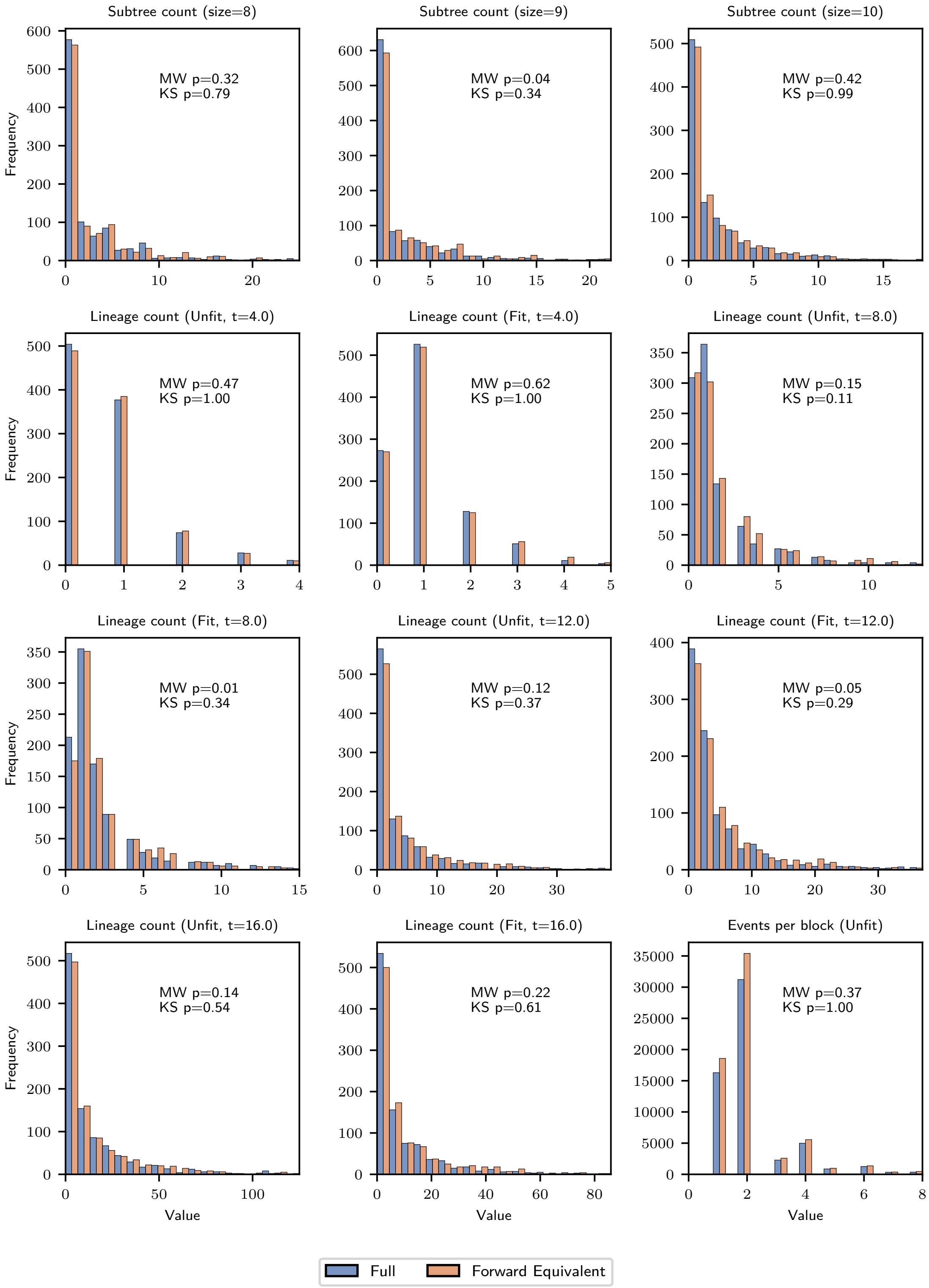

Figure 1: (Continued)

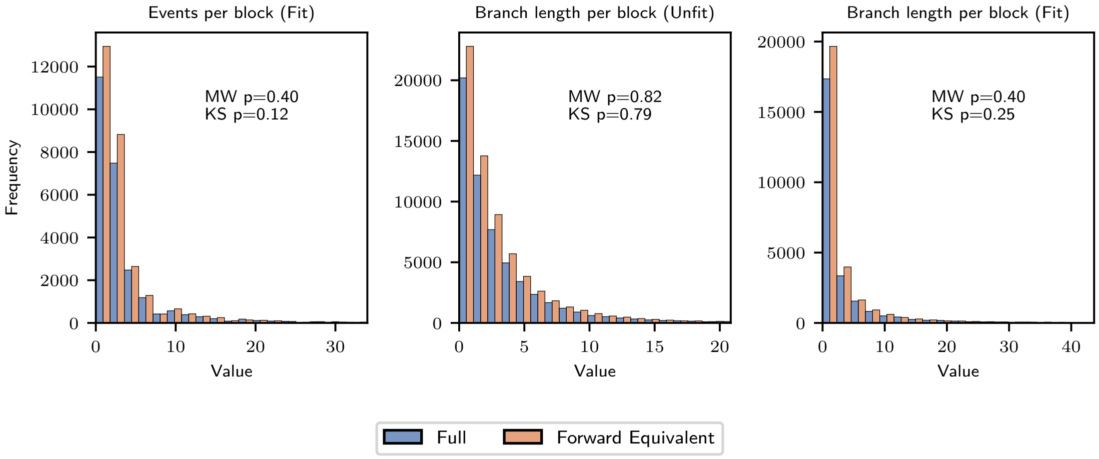

Figure 1: (Continued)

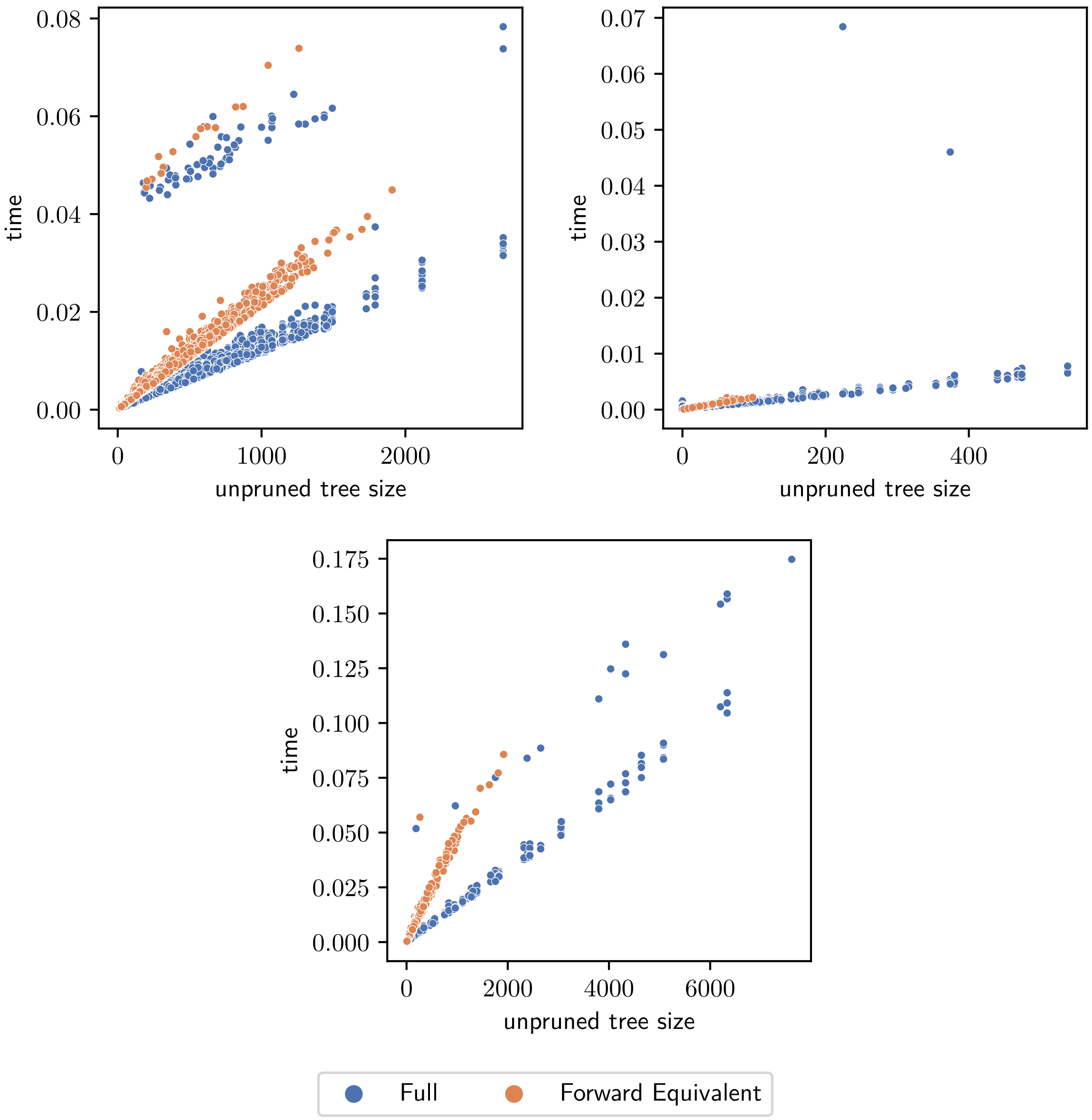

Figure 2: Computation time required for each individual tree against the unpruned tree size across various simulation settings. We see that, in correspondence with the discussion in Sections A and 3.4, the computation time is approximately linear in the size of the unpruned tree. The forward-equivalent model is slower than the full simulation after controlling for the size of the unpruned tree due to the computational overhead of simulating from a BDMS model with time varying rates. The greater efficiency of the forward-equivalent simulation comes from simulating trees with substantially smaller unpruned tree size. Top left: single-type model without death. Top right: single-type model with death. Bottom: multi-type model. Each point is a single tree.
